# Trends in cervical cancer incidence and mortality in the United States, 1975–2018: a population-based study

**DOI:** 10.3389/fmed.2025.1579446

**Published:** 2025-04-30

**Authors:** Xianying Cheng, Ping Wang, Li Cheng, Feng Zhao, Jiangang Liu

**Affiliations:** ^1^Department of Obstetrics and Gynecology, Shulan (Hangzhou) Hospital Affiliated to Zhejiang Shuren University Shulan International Medical College, Hangzhou, Zhejiang, China; ^2^Department of Gynecology, Zouping People’s Hospital, Binzhou, Shandong, China; ^3^Department of Obstetrics and Gynecology, Affiliated Hangzhou Xixi Hospital Zhejiang University School of Medicine, Hangzhou, Zhejiang, China; ^4^Department of Obstetrics and Gynecology, Puren Hospital Affiliated to Wuhan University of Science and Technology, Wuhan, Hubei, China

**Keywords:** cervical cancer, trends, age-adjusted incidence, incidence-based mortality, relative survival

## Abstract

**Background:**

Cervical cancer incidence and mortality rates in the United States have substantially declined over recent decades, primarily driven by reductions in squamous cell carcinoma cases. However, the trend in recent years remains unclear. This study aimed to explore the trends in cervical cancer incidence and mortality, stratified by demographic and tumor characteristics from 1975 to 2018.

**Methods:**

The age-adjusted incidence, incidence-based mortality, and relative survival of cervical cancer were calculated using the Surveillance, Epidemiology, and End Results (SEER)-9 database. Trend analyses with annual percent change (APC) and average annual percent change (AAPC) calculations were performed using Joinpoint Regression Software (Version 4.9.1.0, National Cancer Institute).

**Results:**

During 1975–2018, 49,658 cervical cancer cases were diagnosed, with 17,099 recorded deaths occurring between 1995 and 2018. Squamous cell carcinoma was the most common histological type, with 34,169 cases and 11,859 deaths. Over the study period, the cervical cancer incidence rate decreased by an average of 1.9% (95% CI: −2.3% to −1.6%) per year, with the APCs decreased in recent years (−0.5% [95% CI: −1.1 to 0.1%] in 2006–2018). Squamous cell carcinoma incidence trends closely paralleled overall cervical cancer patterns, but the incidence of squamous cell carcinoma in the distant stage increased significantly (1.1% [95% CI: 0.4 to 1.8%] in 1990–2018). From 1995 to 2018, the overall cervical cancer mortality rate decreased by 1.0% (95% CI: −1.2% to −0.8%) per year. But for distant-stage squamous cell carcinoma, the mortality rate increased by 1.2% (95% CI: 0.3 to 2.1%) per year.

**Conclusion:**

For cervical cancer cases diagnosed in the United States from 1975 to 2018, the overall incidence and mortality rates decreased significantly. However, there was an increase in the incidence and mortality of advanced-stage squamous cell carcinoma. These epidemiological patterns offer critical insights for refining cervical cancer screening protocols and developing targeted interventions for advanced-stage cases.

## 1 Introduction

According to the latest GLOBOCAN statistics, cervical cancer ranks as the fourth most common cancer in terms of incidence and mortality among women worldwide, following only breast cancer, colorectal cancer, and lung cancer (1). In low-income countries, cervical cancer is the second most common fatal malignant tumor (2). Evidently, cervical cancer causes a substantial burden on women’s public health globally.

Cervical carcinogenesis follows a well-defined sequence: (1) normal squamous epithelium, (2) premalignant cervical intraepithelial neoplasia (CIN1-3), and (3) invasive carcinoma (3). Human papillomavirus (HPV) infection is the predominant risk factor for the development of cervical cancer. Almost all cases of cervical cancer are associated with HPV infection, especially HPV-16 and HPV-18. Because of significant advances in understanding the infectious etiology of cervical cancer, HPV vaccines indicated the promising efficacy for cervical cancer prevention (4). The World Health Organization (WHO) launched its 90–70-90 elimination strategy in 2020: 90% HPV vaccination coverage by age 15, 70% screening with high-performance tests by age 35/45, and 90% treatment of precancerous lesions (5). While these initiatives show promise for reducing disease burden, longitudinal epidemiological trend analyses remain scarce.

The global implementation of prevention strategies has demonstrated partial success, with measurable outcomes observed in specific regions. Longitudinal analysis of GLOBOCAN 2018 data (6) revealed stabilized or declining incidence rates and mortality rates in 31 countries. Contrastingly, analysis of Puerto Rico Central Cancer Registry data (2001–2017) by Ortiz et al. (7) indicated a rising incidence specifically among women aged 35–64 years. Cervical cancer epidemiology shows strong correlation with national socioeconomic indicators, particularly healthcare expenditure and education indices. Current implementation of WHO-recommended prevention programs in low-income and developing countries fall substantially short of elimination targets (2). Effective implementation of the WHO Global Strategy requires real-time epidemiological data. For this purpose, this study collected the most dependable and comprehensive statistics from Surveillance, Epidemiology and End Results (SEER) database and carried out subgroup analyses to calculate the age-adjusted incidence and incidence-based mortality of cervical cancer according to demographic and tumor characteristics. This comprehensive analysis provides critical evidence to prevention and management of cervical cancer.

## 2 Methods

### 2.1 Data sources

Patients diagnosed with cervical cancer from the SEER-9 database between 1975 and 2018 were included in the analysis. The database is based on information from 9 high-quality registries in the U.S. and covers about 10% of the U.S. population. For each patient, demographic and tumor characteristics were extracted. The National Center for Health Statistics (NCHS) collected and maintained both national and SEER-9 mortality data (8). The SEER-9 incidence file was used to calculate age-adjusted incidence rates and relative survival rates (9). The SEER-9 incidence-based mortality file (8) provided incidence-based mortality rates (10). To ensure a maximum patient count and avoid underestimating the mortality in the earliest years, the mortality calculations were restricted to patients who were diagnosed between 1975 and 2018 and died between 1995 and 2018.

### 2.2 Demographic characteristics

The demographic indicators concerned in this study were race, age, family income and rural–urban distribution. All information came from medical records or death certificates. Age groups were divided into 0–19, 20–39, 40–59, 60–79, and 80+ years old, with reference to previous studies (11, 12). Family income was divided into low- and high-income groups based on $75,000.

### 2.3 Tumor characteristics

International Classification of Diseases for Oncology, Third Edition (ICD-O-3) was used to define cervical cancer (topography code: C53) and its histological classification (Supplementary Table 1): squamous cell carcinoma (histologic codes: 8070–8078), adenocarcinoma (8140, 8144, 8255, 8384, 8480, 8481, 8482), adenosquamous carcinoma (8560, 8570) and others. Only patients with malignant behavior (behavior code: /3) were selected. WHO grade was classified into I, II, III and unknown based on the grade code provided by SEER database and were limited to patients diagnosed between 1975 and 2017. SEER historic stage A and Combined Summary Stage classified cancer as localized, regional, distant and unknown. Since 1988, cervical cancer has been classified as stages I–IV and unknown according to the American Joint Committee on Cancer staging system (AJCC stage 3rd for 1988–2003; AJCC stage group 6th for 2004–2015; AJCC stage group 7th for 2016–2017; AJCC stage group 8th for 2018+). Beginning in 1983, patients’ tumor size was recorded in SEER database according to 4 different codes (EOD-4 for 1983–1987; EOD-10 for 1988–2003; CS for 2004–2015; Tumor Size Summary for 2016+).

### 2.4 Statistical analysis

SEER*Stat 8.3.9.2 was used to calculate age-adjusted incidence and incidence-based mortality rates, which were expressed per 100,000/years. All rates were adjusted to 2000 US Std Population. Patients with cancers identified only by autopsy or death certificates were excluded. Trends were quantified by annual percentage changes (APC), average annual percent change (AAPC) and 95% confidence interval (CI). This step was done by the Joinpoint program, version 4.9.0.1. The program takes trend data and fits the simplest joinpoint model that the data allow. This enables the user to test that an apparent change in trend is statistically significant. The process of selecting joinpoints involves modeling methods and model optimization. The modeling method uses the grid search method (GSM), through which all possible interval piecewise function connection points are established, and the corresponding sum of squares errors (SSE) and mean squared errors (MSE) of each possible case are calculated. The network point with minimum MSE is selected as the piecewise function connection point, and the equation parameters are fitted according to the selected connection point and interval function. Permutation test (Monte Carlo method) was used to optimize the model. The program performs permutation tests to select the number of joinpoints. Since fitting all N! possible permutations of the data would take too long, the program takes a Monte Carlo sample of these N! data sets, using a random number generator. Specify the size of the Monte Carlo sample of permuted data sets (13). A two-side *p* < 0.05 was considered statistically significant.

## 3 Results

During 1975–2018, a total of 49,658 cervical cancer cases were recorded in the SEER-9 registry (Table 1). White patients (37,140 cases [74.8%]) made up the majority of the cases. Most cervical cancer patients were diagnosed between 20 and 79 years old. The most common histological type of cervical cancer was squamous cell carcinoma (34,169 cases [68.5%]), followed by adenocarcinoma (7,317 cases [14.7%]) and adenosquamous carcinoma (1,653 cases [3.3%]). Between 1995 and 2018, there are 17,099 cervical cancer deaths. White people (12,359 deaths [72.3%]) and patients with squamous cell carcinoma (11,859 deaths [69.4%]) still predominate. The majority of deaths occurred in people over 40 years old, in metropolitan areas and in those with tumors size unknown.

**Table 1:** Cervical cancer incidence (1975–2018) and incidence-based mortality (1995–2018): the SEER-9 registry database.

Characteristic	Incidence				Incidence-based mortality			
	Cervical cancer		SCC		Cervical cancer		SCC	
	Cases, no. (%)	Rate (95% CI)^a^	Cases, no. (%)	Rate (95% CI)^a^	Deaths, no. (%)^b^	Rate (95% CI)^a^	Deaths, no. (%)^b^	Rate (95% CI)^a^
Overall	49658 (100)	8.66 (8.58–8.73)	34169 (100)	5.95 (5.89–6.01)	17099 (100)	4.45 (4.39–4.52)	11859 (100)	3.09 (3.04–3.15)
Race								
White	37140 (74.8)	8.26 (8.17–8.34)	25029 (73.3)	5.56 (5.49–5.63)	12359 (72.3)	4.08 (4.01–4.15)	8470 (71.4)	2.80 (2.74–2.86)
Black	7525 (15.2)	12.72 (12.42–13.01)	5707 (16.7)	9.54 (9.29–9.79)	2989 (17.5)	8.23 (7.93–8.53)	2207 (18.6)	6.05 (5.79–6.31)
Other	4993 (10.1)	8.42 (8.18–8.66)	3433 (10.0)	5.79 (5.59–5.99)	1751 (10.2)	4.09 (3.90–4.29)	1182 (10.0)	2.79 (2.63–2.95)
Age group, y								
0–19	114 (0.3)	0.07 (0.06–0.09)	38 (0.1)	0.02 (0.02–0.03)	NR^c^	NR^c^	NR^c^	NR^c^
20–39	14289 (32.5)	8.68 (8.53–8.82)	9787 (28.6)	5.94 (5.82–6.06)	978 (5.7)	1.06 (0.99–1.13)	643 (5.4)	0.70 (0.65–0.75)
40–59	14514 (33.0)	14.31 (14.12–14.51)	14002 (41.0)	9.92 (9.76–10.09)	4911 (28.7)	5.12 (4.97–5.26)	3419 (28.8)	3.57 (3.45–3.69)
60–79	12146 (27.6)	14.74 (14.48–15.00)	8542 (25.0)	10.33 (10.12–10.56)	6802 (39.8)	13.85 (13.52–14.19)	4719 (39.8)	9.64 (9.36–9.92)
80+	2946 (6.7)	13.30 (12.82–13.79)	1800 (5.3)	8.15 (7.78–8.54)	4403 (25.8)	29.05 (28.20–29.93)	3077 (25.9)	20.35 (19.63–21.08)
Median household income (1990–2018)								
<$75000	18171 (57.4)	8.08 (7.97–8.21)	12255 (59.2)	5.47 (5.37–5.56)	7367 (59.4)	1.95 (1.91–2.00)	4991 (60.7)	1.33 (1.29–1.37)
≥$75000	13469 (42.6)	7.04 (6.92–7.16)	8446 (40.8)	4.41 (4.32–4.51)	5039 (40.6)	1.33 (1.29–1.36)	3230 (39.3)	0.85 (0.82–0.88)
Rural–Urban (1990–2018)								
Metropolitan	27144 (87.4)	7.49 (7.40–7.58)	17673 (87.1)	4.87 (4.80–4.95)	10475 (86.2)	2.77 (2.72–2.83)	6916 (85.9)	1.84 (1.80–1.88)
Non-metropolitan	3899 (12.6)	8.33 (8.06–8.60)	2624 (12.9)	5.63 (5.42–5.86)	1680 (13.8)	0.44 (0.42–0.46)	1132 (14.1)	0.30 (0.28–0.32)
Histologic type								
Squamous cell carcinoma	34169 (68.5)	5.95 (5.89–6.01)			11859 (69.4)	3.09 (3.04–3.15)		
Adenocarcinoma	7317 (14.7)	1.30 (1.27–1.33)			2146 (12.6)	0.56 (0.53–0.58)		
Adenosquamous carcinoma	1653 (3.3)	0.29 (0.28–0.31)			574 (3.3)	0.15 (0.14–0.17)		
Other	6519 (13.1)	1.12 (1.09–1.15)			2520 (14.7)	0.65 (0.62–0.68)		
Grade (1975–2017)								
I	3960 (8.2)	0.72 (0.70–0.75)	1790 (5.3)	0.32 (0.31–0.34)	941 (5.8)	0.25 (0.24–0.27)	491 (4.3)	0.13 (0.12–0.15)
II	11532 (23.8)	2.09 (2.05–2.13)	8571 (25.6)	1.55 (1.51–1.58)	3802 (23.3)	1.05 (1.02–1.09)	2933 (25.9)	0.81 (0.78–0.84)
III	12699 (26.2)	2.27 (2.23–2.31)	8880 (26.5)	1.59 (1.56–1.63)	5023 (30.8)	1.39 (1.36–1.43)	3392 (29.9)	0.94 (0.91–0.97)
Unknown	20339 (41.9)	3.63 (3.58–3.68)	14268 (42.6)	2.55 (2.51–2.59)	6517 (40.0)	1.76 (1.72–1.81)	4518 (39.9)	1.23 (1.19–1.26)
Stage								
Localized	25248 (50.6)	4.45 (4.39–4.51)	17160 (50.0)	3.01 (2.96–3.06)	6531 (37.8)	1.67 (1.63–1.71)	4681 (39.0)	1.20 (1.17–1.23)
Regional	16330 (32.7)	2.83 (2.79–2.88)	12528 (36.5)	2.17 (2.14–2.21)	6796 (39.3)	1.79 (1.75–1.83)	5148 (42.9)	1.35 (1.32–1.39)
Distant	5084 (10.2)	0.87 (0.85–0.89)	3181 (9.3)	0.55 (0.53–0.57)	2699 (15.6)	0.72 (0.70–0.75)	1586 (13.2)	0.43 (0.41–0.45)
Unknown	3283 (6.6)	0.55 (0.54–0.57)	1465 (4.3)	0.25 (0.24–0.26)	1274 (7.4)	0.32 (0.31–0.34)	573 (4.8)	0.15 (0.13–0.16)
AJCC (1988–2018)								
I	17542 (58.6)	4.05 (3.99–4.11)	11345 (50.5)	2.61 (2.56–2.66)	4184 (31.9)	1.09 (1.06–1.12)	2862 (32.7)	0.75 (0.72–0.78)
II	4528 (15.1)	1.02 (0.99–1.05)	3454 (15.4)	0.78 (0.75–0.80)	2127 (16.2)	0.56 (0.54–0.59)	1630 (18.6)	0.43 (0.41–0.45)
III	1274 (4.3)	1.24 (1.21–1.27)	4150 (18.5)	0.94 (0.91–0.97)	2799 (21.3)	0.75 (0.73–0.78)	2066 (23.6)	0.56 (0.53–0.58)
IV	3855 (12.9)	0.86 (0.83–0.88)	2359 (10.5)	0.53 (0.51–0.55)	2706 (20.6)	0.73 (0.70–0.76)	1626 (18.6)	0.44 (0.42–0.46)
Unknown	2726 (9.1)	0.60 (0.58–0.62)	1143 (5.1)	0.25 (0.24–0.27)	1310 (10.0)	0.34 (0.32–0.35)	557 (6.4)	0.14 (0.13–0.16)
Tumor size (1983–2018)								
<2 cm	6650 (16.7)	1.37 (1.34–1.40)	4405 (16.5)	0.91 (0.88–0.93)	1083 (7.5)	0.28 (0.27–0.30)	738 (7.5)	0.19 (0.18–0.21)
≥2 cm	12926 (32.4)	2.63 (2.59–2.68)	8661 (32.4)	1.76 (1.73–1.80)	5437 (37.4)	1.46 (1.42–1.50)	3662 (37.2)	0.99 (0.95–1.02)
Unknown	20265 (50.9)	4.05 (4.00–4.11)	13663 (51.1)	2.74 (2.69–2.78)	8006 (55.1)	2.07 (2.02–2.12)	5445 (55.3)	1.41 (1.38–1.45)

SEER, surveillance, epidemiology and end results; SCC, squamous cell carcinoma; AJCC, American Joint Committee on Cancer.

^aRates were calculated as number of cases or deaths per 100,000 person-years and age-adjusted to the 2000 US standard population.^

^bNo. of deaths were based on cases diagnosed during 1975–2018.^

^cStatistic suppressed because of fewer than 16 cases or deaths in the time interval.^

In terms of incidence and mortality rates (Table 1), black patients had a higher incidence and mortality rate of cervical cancer than white people, reaching 12.72 and 8.23 per 100,000 person-years, respectively. Incidence and mortality rates generally increased with age, but incidence rates peaked in the 60–79 years group (14.74 per 100,000 person-years) and mortality peaks in the 80+ years group (29.05 per 100,000 person-years). Low-income groups had higher incidence and mortality rates, while non-metropolitan groups had higher incidence and lower mortality rates. According to cancer characteristics, squamous cell carcinoma had a much higher incidence (5.95 per 100,000 person-years) and mortality rate (3.09 per 100,000 person-years) than other histological types. In addition, cervical cancer patients diagnosed as AJCC stage I or larger tumor size, had higher incidence and mortality rates.

Of the 49,658 cervical cancer patients occurring during 1975–2018, 2.3% cannot obtain WHO grade, 39.7% were diagnosed before the availability of AJCC stage starting in 1988, and 19.8% were diagnosed before the availability of tumor size starting in 1983. Of the 17,099 cervical cancer deaths occurring during 1995–2018, 4.8% cannot obtain WHO grade, 23.2% were diagnosed before the availability of AJCC stage starting in 1988, and 15.0% were diagnosed before the availability of tumor size starting in 1983. In the years for which data were available, WHO grade (1975–2017) was unknown for 41.9% of cases and 40.0% of deaths, SEER stage (1975–2018) was unknown for 6.6% of cases and 7.4% of deaths, AJCC stage (1988–2018) was unknown for 9.1% of cases and 10.0% of deaths, and tumor size (1983–2018) was unknown for 50.9% of cases and 55.1% of deaths.

**Figure 1:**
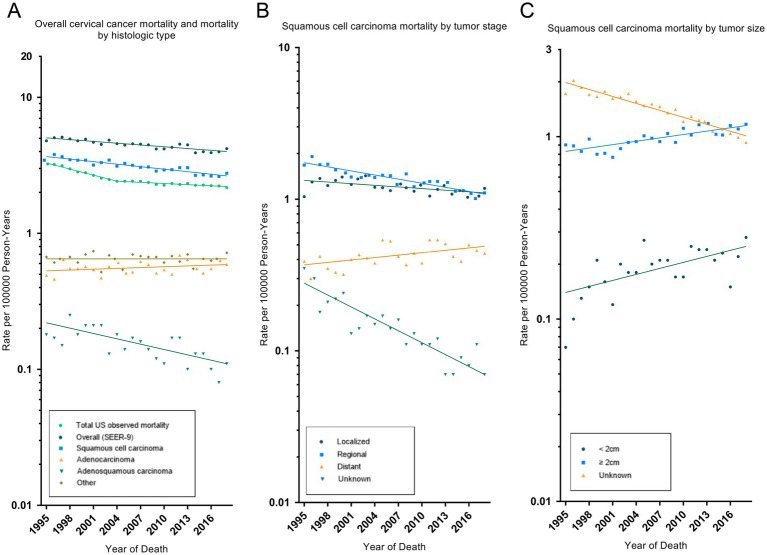
Trends in cervical cancer age-adjusted incidence rates. Panel **(A)** shows overall cervical cancer incidence and incidence by histologic type in 1975–2018. Panel **(B)** shows squamous cell carcinoma incidence by tumor stage in 1975–2018. Panel **(C)** shows squamous cell carcinoma incidence by tumor size in 1983–2018. All rates were age-adjusted to the 2000 US standard population. The dots represent annual incidence rates and the lines represent incidence trends from joinpoint analysis.

Table 2 showed the overall trend of cervical cancer incidence from 1975 to 2018 and the incidence of significant changes in the segments divided by Joinpoint. Figure 1 depicted trends in cervical cancer incidence according to histological type, tumor stage, and tumor size. Cervical cancer incidence rate declined by an average of 1.9% (95% CI, −2.3% to −1.6%) per year over the study period. Among them, the overall incidence rate decreased by −4.7% (95% CI, −5.8% to −3.5%) and −2.7% (95% CI, −3.0% to −2.3%) per year in 1975–1982 and 1990–2006, respectively. However, there was no significant downward trend (APC, −0.5% [95% CI, −1.1 to 0.1%]) between 2006 and 2018. Cervical cancer incidence decreased significantly for all race, age groups, household income and rural–urban distribution. The incidence of squamous cell carcinoma (AAPC, −2.4% [95% CI, −2.8% to −2.0%]) and adenosquamous carcinoma (AAPC, −2.1% [95% CI, −3.0% to −1.2%]) has decreased significantly, while the incidence of adenocarcinoma (AAPC, 1.4% [95% CI, 0.8 to 2.0%]) has increased significantly. SCC incidence decreased significantly for cancer with WHO grade II, III and localized, regional tumor stage. But, the incidence of grade I (2004–2017: APC, 3.9% [95% CI, 1.7 to 6.1%]) and distant metastasized (1990–2018: APC, 1.1% [95% CI, 0.4 to 1.8%]) cases had increased significantly in recent years. The incidence of AJCC stages declined significantly in all SCC, except for stage IV, where there was no significant difference. The incidence of tumor size ≥2 cm increased significantly, mainly due to a significant decrease in the incidence of unknown tumor size.

**Table 2:** Trends in cervical cancer incidence rates (1975–2018): the SEER-9 registry database.

Characteristic	Overall (1975–2018)		Trend^a^														
			1			2			3			4			5		
	AAPC (95% CI)	*P*-value	Year	APC (95% CI)	*P*-value	Year	APC (95% CI)	*P*-value	Year	APC (95% CI)	*P*-value	Year	APC (95% CI)	*P-*value	Year	APC (95% CI)	*P*-value
Overall	−1.9 (−2.3 to −1.6)	<0.001	1975–1982	−4.7 (−5.8 to −3.5)	<0.001	1982–1990	−0.1 (−1.3 to 1.2)	0.911	1990–2006	−2.7 (−3.0 to −2.3)	<0.001	2006–2018	−0.5 (−1.1 to 0.1)	0.101			
Race																	
White	−1.6 (−2.0 to −1.2)	<0.001	1975–1982	−4.8 (−6.1 to −3.5)	<0.001	1982–1990	0.5 (−0.9 to 1.9)	0.508	1990–2005	−2.5 (−2.9 to −2.0)	<0.001	2005–2018	−0.2 (−0.8 to 0.4)	0.582			
Black	−3.0 (−3.8 to −2.3)	<0.001	1975–2015	−3.6 (−3.8 to −3.4)	<0.001	2015–2018	5.2 (−5.8 to 17.5)	0.358									
Other	−2.1 (−2.8 to −1.3)	<0.001	1975–1996	1.4 (−2.1 to −0.6)	0.001	1996–2006	−5.5 (−7.7 to −3.2)	<0.001	2006–2018	−0.5 (−2.0 to 1.0)	0.514						
Age group, y																	
0–19	NR^b^	NR^b^															
20–39	−1.5 (−2.6 to −0.4)	0.010	1975–1981	−4.2 (−6.7 to −1.6)	0.003	1981–1997	−0.3 (−1.0 to 0.3)	0.290	1997–2000	−6.5 (−19.6 to 8.7)	0.369	2000–2018	−0.7 (−1.2 to −0.1)	0.014			
40–59	−1.5 (−2.0 to −1.0)	<0.001	1975–1981	−4.4 (−6.5 to −2.3)	<0.001	1981–1996	−0.7 (−1.4 to −0.1)	0.029	1996–2005	−3.7 (−5.0 to −2.4)	<0.001	2005–2018	0.5 (−0.2 to 1.2)	0.199			
60–79	−2.7 (−2.9 to −2.5)	<0.001															
80+	−3.6 (−3.9 to −3.3)	<0.001															
Median household income (1990–2018)																	
<$75000	−1.6 (−2.0 to −1.2)	<0.001	1990–2010	−2.5 (−2.8 to −2.2)	<0.001	2010–2018	0.7 (−0.6 to 2.0)	0.275									
≥ $75000	−1.7 (−3.0 to −0.4)	0.009	1990–1997	−0.9 (−2.5 to 0.8)	0.275	1997–2000	−7.1 (−17.7 to 4.9)	0.221	2000–2018	−1.1 (−1.6 to −0.7)	<0.001						
Rural–Urban (1990–2018)																	
Metropolitan	−1.7 (−2.1 to −1.3)	<0.001	1990–1997	−1.8 (−2.8 to −0.7)	0.002	1997–2004	−3.8 (−5.1 to −2.5)	<0.001	2004–2018	−0.6 (−1.0 to −0.2)	0.005						
Non-metropolitan	−1.1 (−1.8 to −0.4)	0.002	1990–2007	−2.1 (−2.8 to −1.4)	<0.001	2007–2018	0.4 (−1.2 to 2.1)	0.590									
Histologic type																	
Squamous cell carcinoma	−2.4 (−2.8 to −2.0)	<0.001	1975–1981	−4.5 (−6.0 to −2.9)	<0.001	1981–1997	−1.9 (−2.3 to −1.5)	<0.001	1997–2005	−4.4 (−5.8 to −3.1)	<0.001	2005–2018	−0.8 (−1.4 to −0.2)	0.012			
Adenocarcinoma	1.4 (0.8 to 2.0)	<0.001	1975–1990	2.7 (1.5 to 3.8)	<0.001	1990–2005	−0.5 (−1.5 to 0.5)	0.349	2005–2018	2.2 (1.1 to 3.2)	<0.001						
Adenosquamous carcinoma	−2.1 (−3.0 to −1.2)	<0.001	1975–1996	1.4 (0.1 to 2.7)	0.032	1996–2018	−5.4 (−6.6 to −4.1)	<0.001									
Other	−2.4 (−2.9 to −1.9)	<0.001	1975–1981	−8.8 (−11.7 to −5.7)	<0.001	1981–2018	−1.3 (−1.6 to −1.1)	<0.001									
Squamous cell carcinoma																	
Grade (1975–2017)																	
I	−1.5 (−3.3 to −0.4)	0.119	1975–1991	−5.4 (−6.8 to −4.0)	<0.001	1991–1998	4.1 (−2.8 to 11.4)	0.242	1998–2004	−8.3 (−16.5 to 0.7)	0.068	2004–2017	3.9 (1.7 to 6.1)	0.001			
II	−0.4 (−0.6 to −0.2)	<0.001															
III	−2.1 (−2.7 to −1.5)	<0.001	1975–1981	−6.2 (−9.1 to −3.2)	<0.001	1981–1992	0.8 (−0.6 to 2.3)	0.265	1992–2017	−2.4 (−2.8 to −2.1)	<0.001						
Unknown	−4.3 (−5.7 to −2.8)	<0.001	1975–1998	−3.8 (−4.2 to −3.5)	<0.001	1998–2001	−11.6 (−28.3 to 9.0)	0.239	2001–2017	3.4 (−4.4 to −2.5)	<0.001						
Stage																	
Localized	−2.9 (−3.6 to −2.3)	<0.001	1975–1983	−6.0 (−7.5 to −4.5)	<0.001	1983–1998	−1.0 (−1.7 to −0.3)	0.009	1998–2004	−7.5 (−10.8 to −4.0)	<0.001	2004–2018	−1.2 (−2.1 to −0.3)	0.012			
Regional	−2.0 (−2.1 to −1.8)	<0.001															
Distant	−0.6 (−2.6 to 1.4)	0.552	1975–1986	0.6 (−1.9 to 3.3)	0.623	1986–1990	−14.7 (−30.5 to 4.7)	0.124	1990–2018	1.1 (0.4 to 1.8)	0.002						
Unknown	−5.5 (−10.2 to −0.6)	0.030	1975–1980	−26.7 (−34.5 to −18.0)	<0.001	1980–1984	29.1 (−3.8 to 73.2)	0.086	1984–1998	−4.4 (−6.6 to −2.0)	0.001	1998–2001	−25.9 (−59.0 to 33.8)	0.308	2001–2018	−2.1 (−4.7 to 0.6)	0.125
AJCC (1988–2018)																	
I	−2.8 (−3.9 to −1.7)	<0.001	1988–1998	−1.7 (−3.1 to −0.2)	0.030	1998–2004	−7.5 (−11.8 to −3.0)	0.002	2004–2018	−1.5 (−2.6 to −0.3)	0.016						
II	−1.5 (−2.7 to −0.3)	0.018	1988–2016	−3.4 (−3.7 to −3.1)	<0.001	2016–2018	29.1 (6.6 to 56.4)	0.011									
III	−2.7 (−4.5 to −0.9)	0.004	1988–2006	−1.8 (−2.6 to −1.1)	<0.001	2006–2016	2.0 (0.0 to 4.2)	0.055	2016–2018	−29.2 (−46.0 to −7.2)	0.015						
IV	−0.1 (−0.7 to 0.4)	0.621															
Unknown	−2.6 (−3.9 to −1.3)	<0.001															
Tumor size (1983–2018)																	
<2 cm	1.3 (0.6 to 3.2)	0.170	1983–1993	1.2 (−1.7 to 4.2)	0.412	1993–1997	11.4 (−4.1 to 29.3)	0.151	1997–2018	−0.4 (−1.1 to 0.2)	0.184						
≥2 cm	1.5 (0.8 to 2.2)	<0.001	1983–1989	6.3 (2.9 to 9.8)	0.001	1989–2000	−0.7 (−1.9 to 0.6)	0.269	2000–2018	1.3 (0.8 to 1.7)	<0.001						
Unknown	−5.5 (−6.1 to −4.8)	<0.001	1983–1996	−3.9 (−4.5 to −3.2)	<0.001	1996–2006	−8.5 (−9.9 to −7.1)	<0.001	2006–2018	−4.6 (−5.9 to −3.2)	<0.001						

SEER, surveillance, epidemiology and end results; AJCC, American Joint Committee on Cancer; APC, annual percent change; AAPC, average annual percent change.

^aEach segment was defined based on the identification of years when a statistically significant change in the APC occurred.^

^bAPC could not be calculated because there were 0 deaths in 1 or more years.^

**Figure 2:**
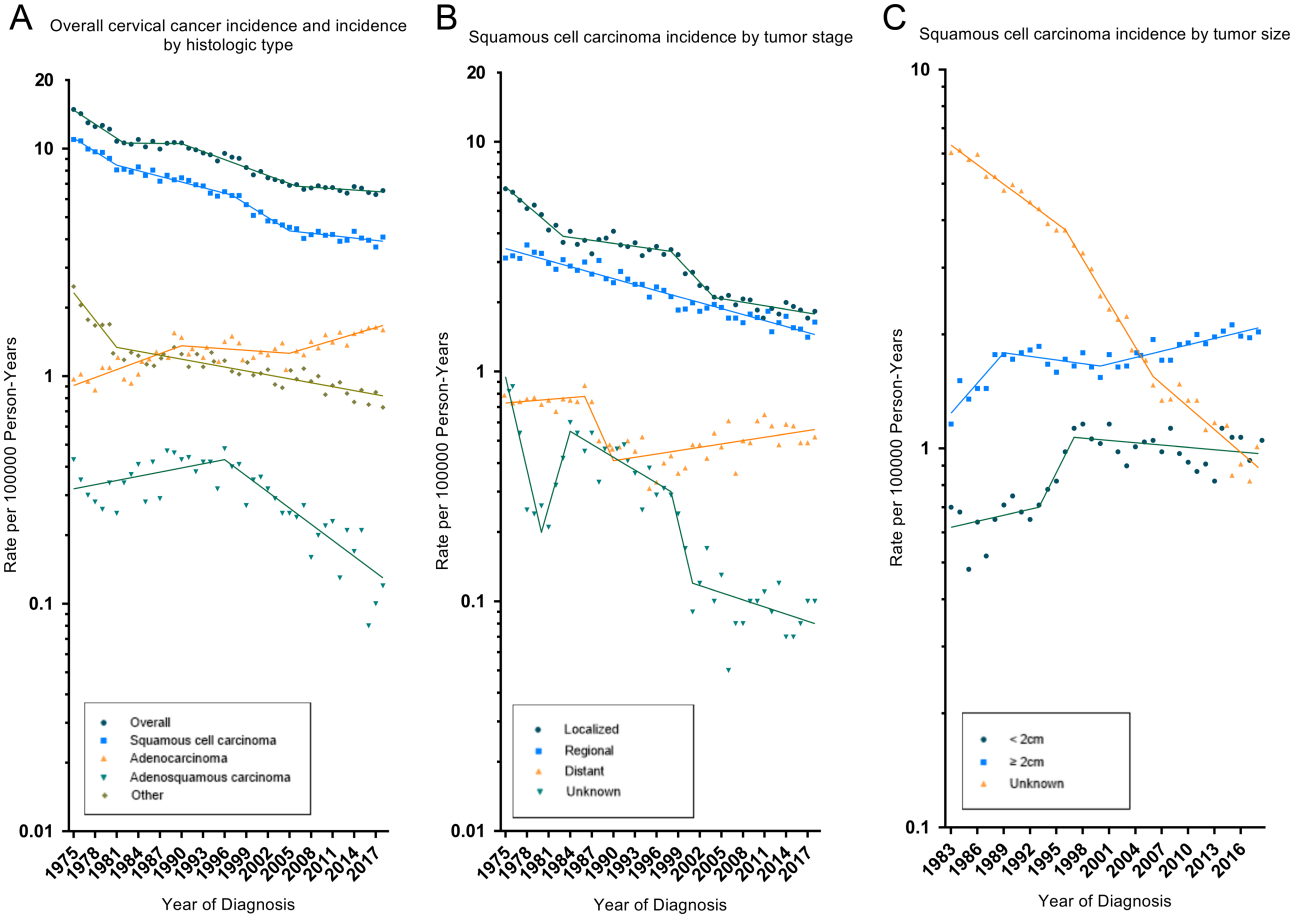
Trends in cervical cancer incidence-based mortality rates. Panel **(A)** shows observed US cervical cancer mortality (1995–2018), SEER-9 cervical cancer incidence-based mortality overall (1995–2018) and by histologic type. Panel **(B)** shows squamous cell carcinoma mortality by tumor stage. Panel **(C)** shows squamous cell carcinoma mortality by tumor size. All rates were age-adjusted to the 2000 US standard population. The dots represent annual mortality rates and the lines represent mortality trends from joinpoint analysis.

During 1995–2018, trends in cervical cancer incidence-based mortality recorded by SEER-9 were consistent with the observed total US cervical cancer mortality (Table 3 and Figure 2). The total US cervical cancer mortality rate declined by 1.7% (95% CI, −1.9% to −1.6%) per year, and its APC declined, from −3.4% (95% CI, −3.7% to −3.0%) in 1995–2004 to −0.7% (95% CI, −0.9% to −0.5%) in 2004–2018. SEER-9 incidence-based mortality decreased, on average, 1.0% (95% CI, −1.2% to −0.8%) annually from 1995 to 2018. All race and age groups had negative AAPC values, except for the rare number of deaths in the 0–19 age group. Cervical cancer mortality rate had increased significantly in low-income groups, with an AAPC of 0.8% (95% CI, 0.3 to 1.2%). By histologic type, there were significant decrease in mortality rates of SCC (AAPC, −1.4% [95% CI, −1.6% to −1.2%]) and adenosquamous carcinoma (AAPC, −2.9% [95% CI, −4.0% to −1.7%]), while adenocarcinoma mortality increased without statistically significant (AAPC, 0.4% [95% CI, −0.2 to 1.1%]). Negative APCs were observed for SCC of all WHO grade, localized, regional or AJCC II stage. However, SCC patients with distant disease had a significantly increased mortality rate (AAPC, 1.2% [95% CI, 0.3 to 2.1%]).

**Table 3:** Trends in observed cervical cancer mortality rates (Total US) and cervical cancer incidence-based mortality rates (1995–2018): the SEER-9 registry database.

Characteristic			Trend^a^								
	Overall (1995–2018)		1			2			3		
	AAPC (95% CI)	*P*-value	Year	APC (95% CI)	*P*-value	Year	APC (95% CI)	*P-*value	Year	APC (95% CI)	*P*-value
US total cervical cancer deaths	−1.7 (−1.9 to −1.6)	<0.001	1995–2004	−3.4 (−3.7 to −3.0)	<0.001	2004–2018	−0.7 (−0.9 to −0.5)	<0.001			
Overall (SEER-9)	−1.0 (−1.2 to −0.8)	<0.001									
Race											
White	−0.7 (−1.0 to −0.4)	<0.001									
Black	−2.5 (−2.9 to −2.0)	<0.001									
Other	−1.6 (−2.4 to −0.8)	0.001									
Age group, y											
0–19	NR^b^	NR^b^									
20–39	−1.1 (−2.3 to 0.0)	0.049									
40–59	−1.3 (−1.8 to −0.9)	<0.001									
60–79	−1.0 (−1.3 to −0.6)	<0.001									
80+	−0.6 (−1.0 to −0.2)	0.008									
Median household income (1990–2018)											
<$75000	0.8 (0.3 to 1.2)	0.002									
≥$75000	−0.6 (−1.8 to −0.6)	0.333	1995–2000	4.6 (−0.8 to 10.3)	0.093	2000–2018	−2.0 (−2.7 to −1.3)	<0.001			
Rural–Urban (1990–2018)											
Metropolitan	0.3 (0.0–0.6)	0.053									
Non-metropolitan	0.5 (−2.4 to 3.5)	0.726	1995–2006	1.5 (−0.8 to 3.8)	0.179	2006–2016	−3.6 (−6.5 to −0.7)	0.018	2016–2018	17.7 (−13.3 to 59.8)	0.275
Histologic type											
Squamous cell carcinoma	−1.4 (−1.6 to −1.2)	<0.001									
Adenocarcinoma	0.4 (−0.2 to 1.1)	0.160									
Adenosquamous carcinoma	−2.9 (−4.0 to −1.7)	<0.001									
Other	0.0 (−0.5 to 0.6)	0.933									
Squamous cell carcinoma											
Grade (1975–2017)											
I	−3.0 (−4.3 to −1.8)	<0.001									
II	−1.0 (−1.7 to −0.4)	0.004									
III	−1.4 (−1.8 to −1.0)	<0.001									
Unknown	−1.6 (−2.1 to −1.1)	<0.001									
Stage											
Localized	−0.8 (−1.3 to −0.3)	0.002									
Regional	−2.1 (−2.4 to −1.7)	<0.001									
Distant	1.2 (0.3 to 2.1)	0.011									
Unknown	−5.5 (−6.6 to −4.4)	<0.001									
AJCC (1988–2018)											
I	1.2 (−0.2 to 2.7)	0.091	1995–2002	6.6 (2.0 to 11.3)	0.007	2002–2018	−1.0 (−2.0 to 0.0)	0.043			
II	−3.1 (−5.1 to −1.0)	0.004	1995–2015	−1.3 (−2.1 to −0.5)	0.003	2015–2018	−14.4 (−27.4 to 1.1)	0.065			
III	−0.5 (−1.2 to 0.3)	0.190									
IV	0.4 (−0.3 to 1.2)	0.274									
Unknown	−4.2 (−9.5 to 1.4)	0.139	1995–2002	−11.4 (−16.1 to −6.6)	<0.001	2002–2005	16.4 (−25.2 to 81.1)	0.479	2005–2018	−4.4 (−6.6 to −2.2)	0.001
Tumor size (1983–2018)											
<2 cm	2.5 (1.1 to 4.0)	0.002									
≥2 cm	1.5 (1.0 to 1.9)	<0.001									
Unknown	−2.9 (−3.2 to −2.5)	<0.001									

SEER, surveillance, epidemiology and end results; AJCC, American Joint Committee on Cancer; APC, annual percent change; AAPC, average annual percent change.

^aEach segment was defined based on the identification of years when a statistically significant change in the APC occurred.^

^bAPC could not be calculated because there were 0 deaths in 1 or more years.^

Table 4 showed the relative survival rates of cervical cancer and SCC in different years of diagnosis. All cases, deaths, incidence rates and mortality rates in the analysis were recorded in Supplementary Tables 2–17.

**Table 4:** Cervical cancer relative survival according to year of diagnosis.

Cervical cancer relative survival															
Survival time	1975–1979	1980–1984	1985–1989	1990–1994	1995–1999	2000–2004	2005–2009	2010	2011	2012	2013	2014	2015	2016	2017
1-year	86.6%	87.2%	87.0%	87.7%	89.0%	87.0%	87.6%	87.7%	85.1%	86.9%	86.6%	87.0%	88.0%	87.5%	88.0%
2-year	77.8%	77.2%	77.8%	79.4%	81.5%	79.3%	78.7%	79.8%	76.3%	77.7%	79.8%	80.3%	80.8%	80.0%	
3-year	73.2%	72.6%	73.0%	75.1%	77.4%	75.2%	74.5%	73.6%	72.5%	73.7%	74.7%	75.9%	76.3%		
4-year	70.3%	69.5%	70.3%	72.1%	74.7%	72.3%	72.0%	71.2%	69.7%	70.8%	71.3%	72.5%			
5-year	68.3%	67.5%	68.4%	70.3%	73.3%	70.1%	69.9%	69.3%	68.1%	69.2%	68.9%				
6-year	67.1%	65.6%	67.1%	68.9%	72.1%	68.8%	68.5%	68.0%	65.8%	68.0%					
7-year	66.0%	64.0%	65.9%	67.9%	71.1%	67.8%	67.5%	67.1%	65.1%						
8-year	65.0%	62.7%	64.8%	67.1%	70.6%	67.0%	66.7%	65.5%							
9-year	64.3%	62.0%	64.1%	66.1%	69.5%	66.4%	65.9%								
10-year	63.1%	61.3%	63.5%	65.4%	68.7%	65.8%	65.5%								

SCC relative survival															
Survival time	1975–1979	1980–1984	1985–1989	1990–1994	1995–1999	2000–2004	2005–2009	2010	2011	2012	2013	2014	2015	2016	2017
1-year	87.8%	88.0%	87.8%	88.7%	90.0%	87.9%	88.5%	87.8%	86.0%	87.3%	87.5%	86.8%	89.9%	88.3%	88.7%
2-year	78.8%	77.6%	78.0%	80.2%	82.2%	80.2%	79.2%	80.5%	76.8%	77.3%	80.5%	80.2%	82.6%	81.5%	
3-year	73.9%	73.3%	73.0%	75.7%	77.9%	75.9%	74.6%	74.6%	72.2%	73.3%	74.6%	75.7%	77.3%		
4-year	70.9%	69.8%	70.2%	72.6%	75.4%	73.2%	71.8%	71.0%	68.8%	71.4%	70.5%	71.8%			
5-year	68.6%	67.7%	68.1%	70.6%	74.0%	70.6%	69.5%	69.0%	67.3%	69.3%	68.1%				
6-year	67.2%	65.7%	66.6%	69.1%	72.8%	69.3%	67.9%	67.8%	65.1%	67.7%					
7-year	66.1%	64.1%	65.6%	68.3%	71.8%	68.3%	67.0%	66.8%	64.4%						
8-year	65.2%	62.8%	64.4%	67.4%	71.3%	67.6%	66.1%	65.4%							
9-year	64.4%	62.1%	63.7%	66.3%	70.2%	67.0%	65.1%								
10-year	63.1%	61.4%	63.0%	65.5%	69.2%	66.1%	64.5%								

SCC, squamous cell carcinoma.

## 4 Discussion

Cervical cancer created an immense burden on global women’s health because of its high incidence and mortality. However, the current study indicated that there was still a certain gap between WHO global strategy and actual epidemiology of cervical cancer. The SEER-9 database had high-quality follow-up of the population of cancer registries in 9 U.S. states for more than 40 years. Benefiting from these data, the present study analyzed in detail the incidence and mortality trends of cervical cancer based on demographic and tumor characteristics. The main findings in our study were that the incidence of cervical cancer declined overall during the study period, but the rate of decline slowed (APC from −4.7% [95% CI: −5.8% to −3.5%] in 1975–1982 to −2.7% [95% CI: −3.0% to −2.3%] in 1990–2006 to −0.5% [95% CI: −1.1% to −0.1%] in 2006–2018); the incidence and mortality of advanced-stage SCC were significantly increasing (APC of incidence = 1.1% [95% CI: 0.4 to 1.8%] in 1990–2018; AAPC of mortality = 1.2% [95% CI: 0.3 to 2.1%] in 1995–2018). The results of the current research were consistent with the most studies reported in recent years. Many studies demonstrated that incidence in cervical cancer patients showed a stable or even decreasing temporal trend among several countries (6, 14, 15).

Accumulated evidence indicated that a variety of risk factors were associated with the occurrence of cervical cancer, including high-risk HPV infection, age, smoking, childbirth and so on (3, 16, 17). Among all of them, HPV infection was the most important risk factor for cervical cancer, especially HPV16 and HPV18, which were estimated to exist in 50–70% cases and 7–20% cases, respectively (18). Persistent infection with HPV increased the possibility of viral genome integration into the host genome through the environment of genomic instability and finally contributed to the development of cervical cancer and other cancers such as head and neck cancers (19). The recognition of the function of HPV infection as the necessary cause of cervical cancer led to the application of HPV screening (20). With the deepening of clinical research, detection of HPV has undergone a significant shift from mild cytological abnormalities, to co-testing with cytology and HPV, and lately to primary HPV screening (21). It was reported that HPV-based screening exhibited 60–70% greater protection against invasive cervical cancers than cytology (22). Therefore, HPV screening is an effective method to improve the detection rate of cervical cancer (21). This was one important reason to explain for the decreasing incidence of cervical cancer on a global scale.

Correspondingly, we conducted sub-group analyses to calculate the incidence of cervical cancer according to race, age, income, area and histological type. We found that except for the increase in the incidence of adenocarcinoma, prevalence rates were generally decreasing in all sub-groups. One possible explanation for this phenomenon could be that the value of HPV screening on adenocarcinoma was not as large as that on squamous cell carcinoma (23). Adenocarcinoma was considered to involve the deep part of the cervical canal, which makes sampling difficult and ultimately leads to missed diagnosis and misdiagnosis (24). Additional researches were needed to improve the detection rate of the patients with adenocarcinoma.

In fact, universal HPV vaccinations were another important reason for the reduction of cervical cancer. Virus-Like Particle (VLP) vaccines were first produced in 1991 and reported to be safe and immunogenic in humans in 2001 (25). After this breakthrough, the first prophylactic HPV vaccine was licensed in 2006 followed by the approval of bivalent HPV vaccine and monovalent HPV vaccine in 2007 and 2014, respectively. Numerus studies suggested that HPV vaccination should occur in females aged between 9 and 26 years old (26). On a global scale, about 24% of girls aged 9–14 years were under the protection of national HPV program in October 2016 (27). To analyze the incidence of cervical cancer in more detail, we thus conducted a sub-analysis according to the year of survey. The results suggested that there were decreasing trends in the incidence of cervical cancer in 1975–1982 and 1990–2006 while no significance differences in 1982–1990 and 2006–2018. Moreover, incidence of cervical cancer patients aged 20–39 exhibited an obvious reduction in 2000–2018. This may be partly attributed to the application of HPV vaccines. As mentioned before, the vaccinated population was mainly teenagers and young woman.

As for the mortality of cervical cancer, we found that the overall mortality decreased significantly while the mortality in patients with low-income and patients with distant metastasis presented an increase tendency. The decreased mortality could be attributed to increasing awareness, early detection, and treatment of the cervical cancer, resulting in better oncological control. It was reported that girls receiving HPV vaccination before 17 years had 88% lower cervical cancer risk and women vaccinated between the ages of 17 and 30 years exhibited half lower risk of cervical cancer (28). Conversely, the increasing mortality might be explained by the inadequate HPV vaccination in females from low-income areas and insufficient HPV screening programs.

Additionally, patients with HIV infections were more vulnerable to HPV-related diseases than those without HIV infections. Women with HIV infections suffered from a high risk of developing invasive cervical cancer, and it was a result of combination of multi-factors (29). Lower clearance of HPV virus and greater predisposition to HPV infection led to high HPV infections in women with HIV infections (30). Persistent HPV infection can enhance the effect of high-risk HPV through immune inhibition (31). One prospective study found that cervical cancer patients with HIV infection were more likely to die from the malignancy than those without HIV infections (32). Hence, it was important to carry out HPV screening and HPV vaccinations in those women with HIV infections which would be beneficial to reduce the incidence and mortality of cervical cancer.

A study on the global burden of cervical cancer (33) showed that the burden of cervical cancer was inversely associated with the socio-demographic index (SDI). Low SDI regions suffered a more severe burden of cervical cancer (the age-standardized incidence rate was 2.62 times that of high SDI regions, and the age-standardized mortality rate was 5.04 times that of high SDI regions). Sub-Saharan Africa was the region with the heaviest burden of cervical cancer globally. Implementing cervical cancer-related policies in these regions incurred high costs, and organized screening and HPV vaccination programs were always inadequate.

We had to admit there were several significant limitations in our study. Given the descriptive nature of our study, the reliability and accuracy of our findings depended on the authenticity of the data used in the present study. The SEER database does not provide information on risk factors at the individual level, lifestyle-related factors, and methods for detecting cervical cancer. Therefore, we could not precisely evaluate the association between all kinds of risk factors and incidence and mortality of cervical cancer, but can only speculate on possible explanations based on actual observed trends in incidence and mortality of cervical cancer. Additionally, there was a relatively large amount of missing data in the early recording period, especially in tumor size. The quality and completeness of the data improved over time. This would cause an over-estimation of incidence and mortality of cervical cancer in sub-group analysis based on tumor size.

## 5 Conclusion

For cervical cancer cases diagnosed in the United States from 1975 to 2018, the overall incidence and mortality rates decreased significantly. However, there was an increase in the incidence and mortality of advanced-stage squamous cell carcinoma. These epidemiological patterns offer critical insights for refining cervical cancer screening protocols and developing targeted interventions for advanced-stage cases.

## Data Availability

The original contributions presented in the study are included in the article/Supplementary material, further inquiries can be directed to the corresponding author.
