# Supplementary material for: Trends in cervical cancer incidence and mortality in the United States, 1975–2018: a population-based study
Source: Front Med (Lausanne). 2025 Apr 30;12:1579446. doi: 10.3389/fmed.2025.1579446 (PMC12074962; doi:10.3389/fmed.2025.1579446)
Supplement: Supplementary file 1 [file Table_1.docx]

Supplementary Material

**Content**

eTable 1. Define the histological classification of cervical cancer according to International Classification of Disease for Oncology, 3rd edition

eTable 2. Cervical cancer incidence rates during 1975-2018, overall and according to race

eTable 3. Cervical cancer incidence rates during 1975-2018 according to age group

eTable 4. Cervical cancer incidence rates during 1990-2018 according to median household income and rural-urban distribution

eTable 5. Cervical cancer incidence rates during 1975-2018 according to histologic type

eTable 6. Squamous cell carcinoma incidence rates during 1975-2017 according to WHO grade

eTable 7. Squamous cell carcinoma incidence rates during 1975-2018 according to stage

eTable 8. Squamous cell carcinoma incidence rates during 1988-2018 according to AJCC stage

eTable 9. Squamous cell carcinoma incidence rates during 1983-2018 according to tumor size

eTable 10. Observed total US cervical cancer mortality and SEER-9 cervical cancer incidence-based mortality rates during 1995-2018, overall and according to race

eTable 11. Cervical cancer incidence-based mortality rates during 1995-2018 according to age group

eTable 12. Cervical cancer incidence-based mortality rates during 1995-2018 according to median household income and rural-urban distribution

eTable 13. Cervical cancer incidence-based mortality rates during 1995-2018 according to histologic type

eTable 14. Squamous cell carcinoma incidence-based mortality rates during 1995-2017 according to WHO grade

eTable 15. Squamous cell carcinoma incidence-based mortality rates during 1995-2018 according to stage

eTable 16. Squamous cell carcinoma incidence-based mortality rates during 1995-2018 according to AJCC stage

eTable 17. Squamous cell carcinoma incidence-based mortality rates during 1995-2018 according to tumor size

eTable 1. Define the histological classification of cervical cancer according to International Classification of Disease for Oncology, 3rd edition (ICD-O-3)

| Histologic type | ICD-O-3 codes |
| --- | --- |
| Squamous cell carcinoma | 8070, 8071, 8072, 8073, 8074, 8075, 8076, 8077, 8078 |
| Adenocarcinoma | 8140, 8144, 8255, 8384, 8480, 8481, 8482 |
| Adenosquamous carcinoma | 8560, 8570 |

eTable 2. Cervical cancer incidence rates^a^ during 1975-2018, overall and according to race

| Year of diagnosis | Overall | | Race | | | | | |
| --- | --- | --- | --- | --- | --- | --- | --- | --- |
|  |  |  | White | | Black | | Other | |
|  | No. cases | Rate | No. cases | Rate | No. cases | Rate | No. cases | Rate |
| 1975 | 1363 | 14.86 | 1070 | 13.35 | 234 | 33.41 | 59 | 14.46 |
| 1976 | 1335 | 14.24 | 1052 | 12.91 | 214 | 29.51 | 69 | 15.31 |
| 1977 | 1237 | 12.98 | 965 | 11.72 | 210 | 28.27 | 62 | 12.83 |
| 1978 | 1209 | 12.51 | 943 | 11.34 | 184 | 24.44 | 82 | 15.54 |
| 1979 | 1241 | 12.66 | 931 | 10.96 | 225 | 28.95 | 85 | 16.66 |
| 1980 | 1232 | 12.19 | 956 | 10.96 | 187 | 23.45 | 89 | 15.64 |
| 1981 | 1103 | 10.78 | 845 | 9.54 | 187 | 24.09 | 71 | 11.72 |
| 1982 | 1097 | 10.61 | 828 | 9.43 | 184 | 21.95 | 85 | 13.40 |
| 1983 | 1109 | 10.46 | 868 | 9.61 | 163 | 18.85 | 78 | 11.46 |
| 1984 | 1195 | 10.97 | 904 | 9.89 | 194 | 21.04 | 87 | 12.30 |
| 1985 | 1114 | 10.19 | 840 | 9.14 | 183 | 19.16 | 91 | 12.58 |
| 1986 | 1186 | 10.77 | 898 | 9.72 | 172 | 18.39 | 116 | 15.32 |
| 1987 | 1123 | 9.96 | 848 | 8.99 | 175 | 18.87 | 100 | 12.33 |
| 1988 | 1216 | 10.57 | 917 | 9.58 | 192 | 19.07 | 107 | 12.93 |
| 1989 | 1245 | 10.64 | 973 | 10.05 | 166 | 15.55 | 106 | 11.97 |
| 1990 | 1259 | 10.60 | 970 | 9.97 | 189 | 16.89 | 100 | 10.37 |
| 1991 | 1213 | 10.04 | 923 | 9.30 | 180 | 16.37 | 110 | 11.23 |
| 1992 | 1227 | 9.89 | 956 | 9.45 | 165 | 14.05 | 106 | 10.74 |
| 1993 | 1202 | 9.58 | 929 | 9.15 | 165 | 13.84 | 108 | 9.93 |
| 1994 | 1204 | 9.40 | 897 | 8.67 | 169 | 14.18 | 138 | 12.45 |
| 1995 | 1147 | 8.83 | 834 | 8.01 | 174 | 13.73 | 139 | 12.24 |
| 1996 | 1255 | 9.53 | 920 | 8.75 | 185 | 14.72 | 150 | 12.19 |
| 1997 | 1223 | 9.17 | 889 | 8.42 | 185 | 13.92 | 149 | 11.77 |
| 1998 | 1227 | 9.06 | 908 | 8.52 | 186 | 13.27 | 133 | 10.03 |
| 1999 | 1130 | 8.27 | 840 | 7.86 | 187 | 13.66 | 103 | 7.38 |
| 2000 | 1067 | 7.67 | 805 | 7.44 | 147 | 10.09 | 115 | 8.02 |
| 2001 | 1111 | 7.94 | 805 | 7.44 | 160 | 11.28 | 146 | 9.69 |
| 2002 | 1052 | 7.45 | 771 | 7.11 | 158 | 10.47 | 123 | 7.92 |
| 2003 | 1037 | 7.31 | 747 | 6.95 | 170 | 11.00 | 120 | 7.31 |
| 2004 | 1024 | 7.16 | 740 | 6.83 | 173 | 10.92 | 111 | 6.61 |
| 2005 | 989 | 6.90 | 729 | 6.75 | 141 | 8.83 | 119 | 6.98 |
| 2006 | 1007 | 6.95 | 743 | 6.83 | 145 | 8.73 | 119 | 6.63 |
| 2007 | 969 | 6.63 | 711 | 6.56 | 144 | 8.70 | 114 | 6.09 |
| 2008 | 1002 | 6.72 | 721 | 6.51 | 167 | 9.60 | 114 | 6.03 |
| 2009 | 1030 | 6.87 | 759 | 6.90 | 144 | 8.07 | 127 | 6.46 |
| 2010 | 1023 | 6.74 | 747 | 6.72 | 151 | 8.25 | 125 | 6.22 |
| 2011 | 1024 | 6.76 | 755 | 6.83 | 143 | 7.96 | 126 | 5.98 |
| 2012 | 1011 | 6.55 | 724 | 6.44 | 144 | 7.78 | 143 | 6.60 |
| 2013 | 997 | 6.38 | 710 | 6.31 | 151 | 7.98 | 136 | 6.00 |
| 2014 | 1060 | 6.81 | 769 | 6.96 | 134 | 6.77 | 157 | 6.75 |
| 2015 | 1075 | 6.71 | 812 | 7.13 | 134 | 6.28 | 129 | 5.50 |
| 2016 | 1030 | 6.43 | 730 | 6.41 | 155 | 7.54 | 145 | 6.00 |
| 2017 | 1014 | 6.29 | 725 | 6.34 | 142 | 6.99 | 147 | 5.92 |
| 2018 | 1054 | 6.55 | 733 | 6.54 | 167 | 7.96 | 154 | 5.96 |

^a^ Rates were calculated as number of cases per 100,000 person-years and age-adjusted to the 2000 U.S. standard population.

eTable 3. Cervical cancer incidence rates^a^ during 1975-2018 according to age group

| Year of diagnosis | Age groups | | | | | | | | | |
| --- | --- | --- | --- | --- | --- | --- | --- | --- | --- | --- |
|  | 0-19 | | 20-39 | | 40-59 | | 60-79 | | 80+ | |
|  | No. cases | Rate | No. cases | Rate | No. cases | Rate | No. cases | Rate | No. cases | Rate |
| 1975 | - | - | 333 | 12.48 | 532 | 23.54 | 415 | 30.97 | 80 | 29.45 |
| 1976 | - | - | 370 | 13.46 | 523 | 22.48 | 360 | 26.51 | 81 | 29.21 |
| 1977 | - | - | 317 | 10.76 | 468 | 20.64 | 351 | 25.20 | 97 | 33.35 |
| 1978 | - | - | 322 | 10.93 | 428 | 18.95 | 373 | 26.16 | 83 | 27.71 |
| 1979 | - | - | 336 | 10.95 | 459 | 20.30 | 356 | 24.54 | 84 | 27.02 |
| 1980 | - | - | 343 | 10.42 | 426 | 18.94 | 372 | 24.91 | 86 | 26.92 |
| 1981 | - | - | 320 | 9.54 | 386 | 17.15 | 325 | 21.28 | 71 | 21.57 |
| 1982 | - | - | 327 | 9.62 | 386 | 17.20 | 313 | 20.02 | 69 | 20.29 |
| 1983 | - | - | 339 | 9.61 | 401 | 17.25 | 291 | 18.41 | 78 | 22.25 |
| 1984 | - | - | 378 | 10.46 | 399 | 17.27 | 321 | 19.93 | 77 | 21.46 |
| 1985 | - | - | 343 | 9.32 | 390 | 16.69 | 311 | 19.00 | 68 | 18.35 |
| 1986 | - | - | 377 | 10.08 | 401 | 17.38 | 334 | 20.14 | 72 | 19.09 |
| 1987 | - | - | 333 | 8.69 | 398 | 16.47 | 324 | 19.42 | 66 | 16.80 |
| 1988 | - | - | 394 | 10.16 | 433 | 17.54 | 304 | 17.73 | 78 | 19.46 |
| 1989 | - | - | 418 | 10.76 | 445 | 17.49 | 310 | 18.00 | 72 | 17.43 |
| 1990 | - | - | 417 | 10.54 | 466 | 17.86 | 300 | 17.23 | 69 | 16.39 |
| 1991 | - | - | 354 | 8.76 | 456 | 17.02 | 322 | 18.44 | 80 | 18.29 |
| 1992 | - | - | 388 | 9.58 | 450 | 16.05 | 301 | 17.21 | 87 | 19.45 |
| 1993 | - | - | 399 | 9.82 | 459 | 15.96 | 264 | 15.07 | 77 | 16.67 |
| 1994 | - | - | 372 | 9.07 | 456 | 15.22 | 308 | 17.60 | 66 | 13.70 |
| 1995 | - | - | 374 | 9.14 | 462 | 14.92 | 248 | 14.11 | 59 | 11.78 |
| 1996 | - | - | 372 | 9.10 | 521 | 16.20 | 281 | 16.12 | 76 | 14.83 |
| 1997 | - | - | 379 | 9.31 | 509 | 15.39 | 263 | 15.11 | 69 | 13.17 |
| 1998 | - | - | 371 | 9.17 | 539 | 15.75 | 237 | 13.58 | 77 | 14.41 |
| 1999 | - | - | 323 | 8.08 | 488 | 13.89 | 249 | 14.18 | 65 | 11.91 |
| 2000 | - | - | 297 | 7.46 | 487 | 13.45 | 199 | 11.35 | 83 | 14.84 |
| 2001 | - | - | 310 | 7.89 | 461 | 12.35 | 263 | 14.98 | 75 | 13.18 |
| 2002 | - | - | 292 | 7.57 | 476 | 12.52 | 215 | 12.08 | 69 | 11.90 |
| 2003 | - | - | 290 | 7.58 | 473 | 12.32 | 211 | 11.72 | 62 | 10.49 |
| 2004 | - | - | 285 | 7.55 | 470 | 12.01 | 207 | 11.28 | 57 | 9.52 |
| 2005 | - | - | 277 | 7.44 | 461 | 11.64 | 193 | 10.50 | 53 | 8.44 |
| 2006 | - | - | 271 | 7.31 | 455 | 11.43 | 224 | 11.75 | 54 | 8.53 |
| 2007 | - | - | 259 | 6.92 | 438 | 10.94 | 223 | 11.48 | 49 | 7.69 |
| 2008 | - | - | 271 | 7.22 | 446 | 11.01 | 229 | 11.12 | 53 | 8.18 |
| 2009 | - | - | 288 | 7.60 | 465 | 11.49 | 223 | 10.59 | 52 | 7.84 |
| 2010 | - | - | 274 | 7.27 | 464 | 11.46 | 225 | 10.15 | 59 | 8.89 |
| 2011 | - | - | 272 | 7.24 | 461 | 11.40 | 238 | 10.71 | 50 | 7.57 |
| 2012 | - | - | 257 | 6.79 | 476 | 11.68 | 227 | 9.64 | 50 | 7.64 |
| 2013 | - | - | 256 | 6.66 | 473 | 11.72 | 211 | 8.37 | 56 | 8.30 |
| 2014 | - | - | 271 | 6.97 | 516 | 12.90 | 223 | 8.81 | 49 | 7.38 |
| 2015 | - | - | 267 | 6.72 | 519 | 12.86 | 252 | 9.31 | 36 | 5.05 |
| 2016 | - | - | 283 | 7.04 | 443 | 11.08 | 247 | 9.15 | 56 | 8.43 |
| 2017 | - | - | 276 | 6.75 | 430 | 10.99 | 256 | 9.19 | 52 | 7.69 |
| 2018 | - | - | 294 | 7.15 | 468 | 11.97 | 247 | 8.58 | 44 | 6.38 |

^a^ Rates were calculated as number of cases per 100,000 person-years and age-adjusted to the 2000 U.S. standard population.

-Statistic suppressed due to <16 cases in the time interval.

eTable 4. Cervical cancer incidence rates^a^ during 1990-2018 according to median household income and rural-urban distribution

| Year of diagnosis | Median household income | | | | Rural-urban distribution | | | |
| --- | --- | --- | --- | --- | --- | --- | --- | --- |
|  | < $75000 | | ≥ $75000 | | Metropolitan | | Non-metropolitan | |
|  | No. cases | Rate | No. cases | Rate | No. cases | Rate | No. cases | Rate |
| 1990 | 724 | 11.17 | 533 | 9.88 | 1036 | 10.62 | 160 | 10.47 |
| 1991 | 726 | 10.95 | 486 | 8.91 | 975 | 9.89 | 176 | 10.84 |
| 1992 | 721 | 10.58 | 506 | 9.06 | 990 | 9.71 | 174 | 10.61 |
| 1993 | 730 | 10.60 | 472 | 8.35 | 991 | 9.61 | 167 | 10.31 |
| 1994 | 702 | 9.94 | 502 | 8.72 | 977 | 9.30 | 159 | 9.50 |
| 1995 | 651 | 9.05 | 495 | 8.54 | 961 | 8.97 | 132 | 7.83 |
| 1996 | 598 | 9.97 | 654 | 9.14 | 1010 | 9.36 | 174 | 10.01 |
| 1997 | 597 | 9.91 | 626 | 8.59 | 1014 | 9.27 | 159 | 9.14 |
| 1998 | 606 | 9.96 | 619 | 8.34 | 1029 | 9.01 | 127 | 8.59 |
| 1999 | 562 | 9.17 | 568 | 7.55 | 943 | 8.19 | 127 | 8.72 |
| 2000 | 536 | 8.47 | 530 | 7.00 | 933 | 7.63 | 133 | 7.91 |
| 2001 | 568 | 8.95 | 541 | 7.10 | 967 | 7.88 | 142 | 8.62 |
| 2002 | 530 | 8.23 | 521 | 6.78 | 897 | 7.22 | 154 | 9.09 |
| 2003 | 511 | 7.92 | 526 | 6.81 | 907 | 7.28 | 130 | 7.58 |
| 2004 | 499 | 7.70 | 523 | 6.71 | 886 | 7.04 | 136 | 8.03 |
| 2005 | 524 | 8.08 | 464 | 5.92 | 860 | 6.82 | 128 | 7.50 |
| 2006 | 619 | 7.44 | 387 | 6.26 | 876 | 6.88 | 130 | 7.71 |
| 2007 | 574 | 6.92 | 395 | 6.23 | 854 | 6.63 | 115 | 6.68 |
| 2008 | 602 | 7.07 | 396 | 6.20 | 884 | 6.63 | 114 | 7.33 |
| 2009 | 659 | 7.33 | 370 | 6.15 | 910 | 6.78 | 119 | 7.85 |
| 2010 | 658 | 6.76 | 365 | 6.71 | 892 | 6.54 | 131 | 8.78 |
| 2011 | 692 | 7.06 | 331 | 6.19 | 912 | 6.72 | 111 | 7.33 |
| 2012 | 693 | 6.68 | 318 | 6.27 | 901 | 6.48 | 110 | 7.44 |
| 2013 | 667 | 6.57 | 330 | 6.04 | 891 | 6.38 | 106 | 6.36 |
| 2014 | 712 | 7.14 | 348 | 6.25 | 943 | 6.70 | 117 | 8.21 |
| 2015 | 650 | 7.13 | 425 | 6.17 | 961 | 6.66 | 114 | 7.42 |
| 2016 | 633 | 6.98 | 397 | 5.71 | 909 | 6.27 | 121 | 8.07 |
| 2017 | 616 | 7.17 | 398 | 5.30 | 894 | 6.16 | 120 | 7.45 |
| 2018 | 611 | 7.30 | 443 | 5.73 | 941 | 6.46 | 113 | 7.68 |

^a^ Rates were calculated as number of cases per 100,000 person-years and age-adjusted to the 2000 U.S. standard population.

eTable 5. Cervical cancer incidence rates^a^ during 1975-2018 according to histologic type

| Year of diagnosis | Histologic type | | | | | | | |
| --- | --- | --- | --- | --- | --- | --- | --- | --- |
|  | Squamous cell carcinoma | | Adenocarcinoma | | Adenosquamous carcinoma | | Other | |
|  | No. cases | Rate | No. cases | Rate | No. cases | Rate | No. cases | Rate |
| 1975 | 1010 | 10.98 | 88 | 0.98 | 37 | 0.43 | 228 | 2.48 |
| 1976 | 1010 | 10.82 | 94 | 1.03 | 31 | 0.35 | 195 | 2.05 |
| 1977 | 951 | 9.96 | 89 | 0.95 | 27 | 0.30 | 170 | 1.77 |
| 1978 | 933 | 9.69 | 83 | 0.87 | 27 | 0.29 | 166 | 1.67 |
| 1979 | 945 | 9.62 | 107 | 1.09 | 24 | 0.26 | 165 | 1.68 |
| 1980 | 923 | 9.07 | 105 | 1.09 | 30 | 0.34 | 174 | 1.69 |
| 1981 | 824 | 8.06 | 119 | 1.21 | 25 | 0.25 | 135 | 1.26 |
| 1982 | 839 | 8.12 | 98 | 0.97 | 34 | 0.34 | 126 | 1.18 |
| 1983 | 841 | 7.89 | 98 | 0.93 | 37 | 0.37 | 133 | 1.27 |
| 1984 | 898 | 8.31 | 106 | 1.02 | 43 | 0.41 | 138 | 1.23 |
| 1985 | 839 | 7.63 | 124 | 1.16 | 28 | 0.28 | 123 | 1.13 |
| 1986 | 891 | 8.05 | 125 | 1.19 | 44 | 0.42 | 126 | 1.11 |
| 1987 | 809 | 7.19 | 141 | 1.28 | 33 | 0.29 | 140 | 1.20 |
| 1988 | 881 | 7.64 | 132 | 1.21 | 56 | 0.47 | 147 | 1.25 |
| 1989 | 855 | 7.29 | 179 | 1.55 | 53 | 0.46 | 158 | 1.34 |
| 1990 | 883 | 7.44 | 174 | 1.48 | 52 | 0.43 | 150 | 1.25 |
| 1991 | 871 | 7.25 | 152 | 1.25 | 54 | 0.44 | 136 | 1.10 |
| 1992 | 858 | 6.94 | 162 | 1.32 | 47 | 0.38 | 160 | 1.25 |
| 1993 | 859 | 6.85 | 149 | 1.21 | 53 | 0.42 | 141 | 1.10 |
| 1994 | 814 | 6.37 | 174 | 1.34 | 54 | 0.42 | 162 | 1.27 |
| 1995 | 804 | 6.19 | 150 | 1.16 | 41 | 0.32 | 152 | 1.16 |
| 1996 | 848 | 6.47 | 187 | 1.42 | 63 | 0.48 | 157 | 1.17 |
| 1997 | 827 | 6.22 | 200 | 1.51 | 54 | 0.40 | 142 | 1.05 |
| 1998 | 840 | 6.23 | 189 | 1.40 | 56 | 0.41 | 142 | 1.03 |
| 1999 | 774 | 5.68 | 161 | 1.18 | 36 | 0.27 | 159 | 1.15 |
| 2000 | 707 | 5.10 | 168 | 1.21 | 49 | 0.35 | 143 | 1.01 |
| 2001 | 736 | 5.27 | 179 | 1.28 | 50 | 0.36 | 146 | 1.03 |
| 2002 | 679 | 4.81 | 175 | 1.24 | 45 | 0.32 | 153 | 1.07 |
| 2003 | 677 | 4.78 | 188 | 1.32 | 40 | 0.29 | 132 | 0.92 |
| 2004 | 659 | 4.62 | 200 | 1.40 | 36 | 0.25 | 129 | 0.89 |
| 2005 | 646 | 4.51 | 152 | 1.08 | 37 | 0.25 | 154 | 1.06 |
| 2006 | 646 | 4.45 | 184 | 1.29 | 34 | 0.24 | 143 | 0.97 |
| 2007 | 592 | 4.03 | 175 | 1.24 | 40 | 0.27 | 162 | 1.08 |
| 2008 | 625 | 4.19 | 205 | 1.42 | 25 | 0.16 | 147 | 0.96 |
| 2009 | 652 | 4.33 | 195 | 1.33 | 31 | 0.20 | 152 | 1.01 |
| 2010 | 632 | 4.16 | 226 | 1.52 | 33 | 0.22 | 132 | 0.83 |
| 2011 | 639 | 4.20 | 207 | 1.41 | 34 | 0.23 | 144 | 0.91 |
| 2012 | 608 | 3.92 | 235 | 1.57 | 20 | 0.13 | 148 | 0.94 |
| 2013 | 616 | 3.96 | 212 | 1.37 | 33 | 0.21 | 136 | 0.84 |
| 2014 | 676 | 4.33 | 231 | 1.54 | 26 | 0.17 | 127 | 0.77 |
| 2015 | 652 | 4.05 | 245 | 1.58 | 32 | 0.21 | 146 | 0.87 |
| 2016 | 638 | 3.96 | 252 | 1.63 | - | - | 127 | 0.75 |
| 2017 | 597 | 3.70 | 253 | 1.64 | 17 | 0.10 | 147 | 0.85 |
| 2018 | 660 | 4.09 | 249 | 1.60 | 19 | 0.12 | 126 | 0.73 |

^a^ Rates were calculated as number of cases per 100,000 person-years and age-adjusted to the 2000 U.S. standard population.

-Statistic suppressed due to <16 cases in the time interval.

eTable 6. Squamous cell carcinoma incidence rates^a^ during 1975-2017 according to WHO grade

| Year of diagnosis | WHO grade | | | | | | | |
| --- | --- | --- | --- | --- | --- | --- | --- | --- |
|  | I | | II | | III | | Unknown | |
|  | No. cases | Rate | No. cases | Rate | No. cases | Rate | No. cases | Rate |
| 1975 | 62 | 0.68 | 132 | 1.39 | 236 | 2.58 | 580 | 6.33 |
| 1976 | 55 | 0.58 | 161 | 1.72 | 222 | 2.39 | 577 | 6.14 |
| 1977 | 53 | 0.56 | 187 | 1.97 | 228 | 2.40 | 483 | 5.03 |
| 1978 | 59 | 0.64 | 167 | 1.74 | 216 | 2.22 | 491 | 5.09 |
| 1979 | 49 | 0.49 | 170 | 1.78 | 193 | 1.92 | 533 | 5.44 |
| 1980 | 54 | 0.52 | 159 | 1.56 | 187 | 1.89 | 523 | 5.11 |
| 1981 | 36 | 0.38 | 165 | 1.62 | 174 | 1.71 | 449 | 4.35 |
| 1982 | 36 | 0.35 | 168 | 1.62 | 200 | 1.95 | 435 | 4.20 |
| 1983 | 41 | 0.39 | 189 | 1.79 | 185 | 1.76 | 426 | 3.96 |
| 1984 | 49 | 0.47 | 170 | 1.59 | 183 | 1.74 | 496 | 4.52 |
| 1985 | 47 | 0.43 | 175 | 1.60 | 186 | 1.72 | 431 | 3.88 |
| 1986 | 39 | 0.35 | 193 | 1.76 | 236 | 2.16 | 423 | 3.79 |
| 1987 | 45 | 0.41 | 156 | 1.38 | 188 | 1.71 | 420 | 3.70 |
| 1988 | 30 | 0.27 | 185 | 1.62 | 207 | 1.82 | 459 | 3.94 |
| 1989 | 35 | 0.30 | 181 | 1.56 | 220 | 1.86 | 419 | 3.56 |
| 1990 | 30 | 0.25 | 194 | 1.66 | 228 | 1.91 | 431 | 3.62 |
| 1991 | 31 | 0.25 | 203 | 1.71 | 224 | 1.94 | 413 | 3.35 |
| 1992 | 33 | 0.27 | 198 | 1.60 | 245 | 2.05 | 382 | 3.03 |
| 1993 | 38 | 0.31 | 201 | 1.61 | 246 | 2.02 | 374 | 2.92 |
| 1994 | 43 | 0.33 | 199 | 1.56 | 232 | 1.84 | 340 | 2.64 |
| 1995 | 39 | 0.30 | 188 | 1.45 | 249 | 1.95 | 328 | 2.49 |
| 1996 | 38 | 0.29 | 237 | 1.81 | 231 | 1.77 | 342 | 2.59 |
| 1997 | 48 | 0.36 | 195 | 1.47 | 236 | 1.78 | 348 | 2.61 |
| 1998 | 45 | 0.34 | 224 | 1.66 | 223 | 1.66 | 348 | 2.58 |
| 1999 | 42 | 0.31 | 233 | 1.70 | 203 | 1.51 | 296 | 2.17 |
| 2000 | 42 | 0.30 | 209 | 1.50 | 190 | 1.38 | 266 | 1.91 |
| 2001 | 48 | 0.35 | 238 | 1.71 | 222 | 1.58 | 228 | 1.63 |
| 2002 | 26 | 0.19 | 208 | 1.48 | 210 | 1.47 | 235 | 1.68 |
| 2003 | 33 | 0.23 | 208 | 1.47 | 193 | 1.35 | 243 | 1.73 |
| 2004 | 31 | 0.22 | 190 | 1.33 | 198 | 1.39 | 240 | 1.68 |
| 2005 | 27 | 0.19 | 229 | 1.62 | 195 | 1.33 | 195 | 1.37 |
| 2006 | 40 | 0.28 | 214 | 1.47 | 204 | 1.40 | 188 | 1.30 |
| 2007 | 28 | 0.19 | 178 | 1.23 | 196 | 1.31 | 190 | 1.30 |
| 2008 | 32 | 0.22 | 208 | 1.39 | 190 | 1.26 | 195 | 1.31 |
| 2009 | 40 | 0.27 | 224 | 1.48 | 218 | 1.43 | 170 | 1.14 |
| 2010 | 35 | 0.24 | 229 | 1.53 | 195 | 1.25 | 173 | 1.14 |
| 2011 | 41 | 0.28 | 223 | 1.47 | 194 | 1.27 | 181 | 1.19 |
| 2012 | 40 | 0.27 | 207 | 1.34 | 197 | 1.23 | 164 | 1.08 |
| 2013 | 42 | 0.28 | 238 | 1.53 | 171 | 1.07 | 165 | 1.08 |
| 2014 | 56 | 0.38 | 246 | 1.57 | 189 | 1.22 | 185 | 1.17 |
| 2015 | 50 | 0.30 | 254 | 1.58 | 189 | 1.19 | 159 | 0.98 |
| 2016 | 50 | 0.32 | 226 | 1.44 | 183 | 1.13 | 179 | 1.08 |
| 2017 | 52 | 0.35 | 212 | 1.34 | 168 | 1.00 | 165 | 1.02 |

^a^ Rates were calculated as number of cases per 100,000 person-years and age-adjusted to the 2000 U.S. standard population.

eTable 7. Squamous cell carcinoma incidence rates^a^ during 1975-2018 according to stage

| Year of diagnosis | Stage | | | | | | | |
| --- | --- | --- | --- | --- | --- | --- | --- | --- |
|  | Localized | | Regional | | Distant | | Unknown | |
|  | No. cases | Rate | No. cases | Rate | No. cases | Rate | No. cases | Rate |
| 1975 | 576 | 6.25 | 288 | 3.12 | 71 | 0.79 | 75 | 0.82 |
| 1976 | 565 | 6.04 | 300 | 3.19 | 70 | 0.73 | 80 | 0.86 |
| 1977 | 529 | 5.57 | 300 | 3.11 | 71 | 0.74 | 51 | 0.54 |
| 1978 | 500 | 5.12 | 336 | 3.56 | 73 | 0.76 | 24 | 0.25 |
| 1979 | 531 | 5.31 | 312 | 3.31 | 78 | 0.77 | 24 | 0.24 |
| 1980 | 491 | 4.82 | 330 | 3.27 | 74 | 0.72 | 28 | 0.26 |
| 1981 | 425 | 4.13 | 301 | 2.96 | 77 | 0.75 | 21 | 0.21 |
| 1982 | 449 | 4.33 | 286 | 2.80 | 70 | 0.67 | 34 | 0.32 |
| 1983 | 397 | 3.65 | 320 | 3.07 | 79 | 0.76 | 45 | 0.42 |
| 1984 | 446 | 4.08 | 306 | 2.88 | 81 | 0.75 | 65 | 0.60 |
| 1985 | 396 | 3.58 | 300 | 2.76 | 83 | 0.74 | 60 | 0.54 |
| 1986 | 422 | 3.73 | 324 | 3.00 | 95 | 0.87 | 50 | 0.45 |
| 1987 | 370 | 3.25 | 295 | 2.66 | 82 | 0.74 | 62 | 0.54 |
| 1988 | 449 | 3.76 | 338 | 3.05 | 55 | 0.50 | 39 | 0.33 |
| 1989 | 456 | 3.81 | 293 | 2.54 | 53 | 0.48 | 53 | 0.46 |
| 1990 | 492 | 4.08 | 282 | 2.44 | 54 | 0.46 | 55 | 0.46 |
| 1991 | 439 | 3.56 | 316 | 2.73 | 55 | 0.47 | 61 | 0.48 |
| 1992 | 440 | 3.50 | 307 | 2.53 | 60 | 0.50 | 51 | 0.42 |
| 1993 | 466 | 3.64 | 290 | 2.40 | 56 | 0.45 | 47 | 0.37 |
| 1994 | 420 | 3.20 | 296 | 2.40 | 65 | 0.52 | 33 | 0.25 |
| 1995 | 444 | 3.39 | 271 | 2.11 | 39 | 0.31 | 50 | 0.38 |
| 1996 | 464 | 3.51 | 302 | 2.34 | 43 | 0.33 | 39 | 0.29 |
| 1997 | 437 | 3.24 | 295 | 2.26 | 53 | 0.40 | 42 | 0.31 |
| 1998 | 460 | 3.40 | 282 | 2.12 | 58 | 0.43 | 40 | 0.29 |
| 1999 | 441 | 3.23 | 251 | 1.85 | 48 | 0.36 | 34 | 0.25 |
| 2000 | 371 | 2.67 | 259 | 1.87 | 53 | 0.38 | 24 | 0.17 |
| 2001 | 377 | 2.71 | 279 | 1.99 | 68 | 0.48 | - | - |
| 2002 | 331 | 2.37 | 261 | 1.83 | 69 | 0.48 | 18 | 0.12 |
| 2003 | 323 | 2.31 | 272 | 1.89 | 59 | 0.42 | 23 | 0.17 |
| 2004 | 299 | 2.11 | 280 | 1.96 | 79 | 0.54 | 16 | 0.10 |
| 2005 | 287 | 2.09 | 280 | 1.90 | 70 | 0.47 | 20 | 0.13 |
| 2006 | 304 | 2.15 | 255 | 1.71 | 90 | 0.61 | - | - |
| 2007 | 277 | 1.95 | 258 | 1.71 | 56 | 0.36 | - | - |
| 2008 | 303 | 2.07 | 246 | 1.63 | 77 | 0.50 | - | - |
| 2009 | 298 | 2.05 | 273 | 1.78 | 80 | 0.49 | - | - |
| 2010 | 270 | 1.85 | 266 | 1.72 | 97 | 0.61 | 17 | 0.10 |
| 2011 | 247 | 1.71 | 285 | 1.83 | 105 | 0.65 | 19 | 0.11 |
| 2012 | 277 | 1.88 | 242 | 1.49 | 93 | 0.58 | - | - |
| 2013 | 263 | 1.78 | 263 | 1.63 | 81 | 0.49 | 20 | 0.12 |
| 2014 | 305 | 2.00 | 276 | 1.74 | 95 | 0.59 | - | - |
| 2015 | 299 | 1.92 | 253 | 1.55 | 102 | 0.58 | - | - |
| 2016 | 285 | 1.85 | 254 | 1.53 | 85 | 0.49 | - | - |
| 2017 | 259 | 1.71 | 236 | 1.41 | 86 | 0.49 | 16 | 0.10 |
| 2018 | 280 | 1.83 | 269 | 1.64 | 93 | 0.52 | 17 | 0.10 |

^a^ Rates were calculated as number of cases per 100,000 person-years and age-adjusted to the 2000 U.S. standard population.

-Statistic suppressed due to <16 cases in the time interval.

eTable 8. Squamous cell carcinoma incidence rates^a^ during 1988-2018 according to AJCC stage

| Year of diagnosis | AJCC stage | | | | | | | | | |
| --- | --- | --- | --- | --- | --- | --- | --- | --- | --- | --- |
|  | I | | II | | III | | IV | | Unknown | |
|  | No. cases | Rate | No. cases | Rate | No. cases | Rate | No. cases | Rate | No. cases | Rate |
| 1988 | 486 | 4.10 | 156 | 1.39 | 131 | 1.20 | 68 | 0.61 | 40 | 0.34 |
| 1989 | 476 | 3.97 | 130 | 1.14 | 127 | 1.10 | 68 | 0.61 | 54 | 0.47 |
| 1990 | 514 | 4.26 | 131 | 1.17 | 119 | 1.02 | 64 | 0.53 | 55 | 0.46 |
| 1991 | 461 | 3.75 | 143 | 1.25 | 141 | 1.21 | 64 | 0.55 | 62 | 0.49 |
| 1992 | 454 | 3.61 | 134 | 1.12 | 141 | 1.16 | 77 | 0.63 | 52 | 0.42 |
| 1993 | 475 | 3.71 | 121 | 1.00 | 142 | 1.17 | 71 | 0.59 | 50 | 0.39 |
| 1994 | 443 | 3.39 | 123 | 0.99 | 136 | 1.11 | 78 | 0.63 | 34 | 0.26 |
| 1995 | 457 | 3.49 | 122 | 0.95 | 122 | 0.95 | 52 | 0.41 | 51 | 0.39 |
| 1996 | 490 | 3.71 | 126 | 0.97 | 133 | 1.03 | 58 | 0.45 | 41 | 0.31 |
| 1997 | 455 | 3.39 | 126 | 0.97 | 131 | 0.99 | 70 | 0.54 | 45 | 0.33 |
| 1998 | 480 | 3.55 | 124 | 0.93 | 128 | 0.96 | 65 | 0.48 | 43 | 0.31 |
| 1999 | 453 | 3.32 | 111 | 0.81 | 112 | 0.83 | 60 | 0.45 | 38 | 0.27 |
| 2000 | 389 | 2.80 | 114 | 0.82 | 117 | 0.84 | 63 | 0.46 | 24 | 0.17 |
| 2001 | 389 | 2.80 | 114 | 0.82 | 138 | 0.98 | 83 | 0.59 | - | - |
| 2002 | 343 | 2.46 | 114 | 0.80 | 125 | 0.88 | 79 | 0.55 | 18 | 0.12 |
| 2003 | 337 | 2.40 | 116 | 0.80 | 130 | 0.92 | 69 | 0.48 | 25 | 0.18 |
| 2004 | 306 | 2.16 | 107 | 0.76 | 131 | 0.92 | 75 | 0.52 | 40 | 0.26 |
| 2005 | 290 | 2.10 | 109 | 0.74 | 135 | 0.93 | 66 | 0.44 | 46 | 0.30 |
| 2006 | 308 | 2.18 | 105 | 0.70 | 121 | 0.83 | 89 | 0.60 | 23 | 0.15 |
| 2007 | 281 | 1.98 | 104 | 0.70 | 124 | 0.82 | 55 | 0.35 | 28 | 0.19 |
| 2008 | 303 | 2.08 | 89 | 0.59 | 122 | 0.80 | 74 | 0.48 | 37 | 0.24 |
| 2009 | 297 | 2.04 | 96 | 0.62 | 148 | 0.97 | 78 | 0.48 | 33 | 0.22 |
| 2010 | 271 | 1.86 | 93 | 0.60 | 147 | 0.96 | 92 | 0.58 | 29 | 0.17 |
| 2011 | 248 | 1.71 | 101 | 0.64 | 158 | 1.04 | 101 | 0.63 | 31 | 0.19 |
| 2012 | 279 | 1.89 | 73 | 0.47 | 138 | 0.84 | 93 | 0.58 | 25 | 0.15 |
| 2013 | 258 | 1.75 | 82 | 0.51 | 161 | 1.00 | 79 | 0.47 | 36 | 0.22 |
| 2014 | 308 | 2.02 | 97 | 0.59 | 159 | 1.02 | 92 | 0.56 | 20 | 0.13 |
| 2015 | 302 | 1.93 | 77 | 0.46 | 151 | 0.95 | 102 | 0.58 | 20 | 0.12 |
| 2016 | 250 | 1.62 | 87 | 0.53 | 167 | 1.00 | 86 | 0.50 | 48 | 0.31 |
| 2017 | 214 | 1.42 | 91 | 0.53 | 135 | 0.83 | 95 | 0.54 | 62 | 0.39 |
| 2018 | 328 | 2.13 | 138 | 0.86 | 80 | 0.47 | 93 | 0.52 | 21 | 0.12 |

Abbreviation: AJCC, American Joint Committee on Cancer

^a^ Rates were calculated as number of cases per 100,000 person-years and age-adjusted to the 2000 U.S. standard population.

-Statistic suppressed due to <16 cases in the time interval.

eTable 9. Squamous cell carcinoma incidence rates^a^ during 1983-2018 according to tumor size

| Year of diagnosis | Tumor size | | | | | |
| --- | --- | --- | --- | --- | --- | --- |
|  | < 2cm | | ≥ 2cm | | Unknown | |
|  | No. cases | Rate | No. cases | Rate | No. cases | Rate |
| 1983 | 75 | 0.70 | 121 | 1.16 | 645 | 6.04 |
| 1984 | 73 | 0.68 | 159 | 1.51 | 666 | 6.13 |
| 1985 | 54 | 0.48 | 148 | 1.35 | 637 | 5.79 |
| 1986 | 78 | 0.64 | 151 | 1.44 | 662 | 5.98 |
| 1987 | 57 | 0.52 | 156 | 1.44 | 596 | 5.23 |
| 1988 | 77 | 0.65 | 202 | 1.77 | 602 | 5.22 |
| 1989 | 87 | 0.72 | 204 | 1.77 | 564 | 4.80 |
| 1990 | 92 | 0.75 | 200 | 1.72 | 591 | 4.97 |
| 1991 | 84 | 0.68 | 206 | 1.79 | 581 | 4.78 |
| 1992 | 83 | 0.65 | 220 | 1.82 | 555 | 4.47 |
| 1993 | 93 | 0.71 | 226 | 1.86 | 540 | 4.29 |
| 1994 | 104 | 0.78 | 208 | 1.67 | 502 | 3.92 |
| 1995 | 109 | 0.83 | 205 | 1.59 | 490 | 3.77 |
| 1996 | 130 | 0.98 | 222 | 1.72 | 496 | 3.77 |
| 1997 | 151 | 1.13 | 215 | 1.65 | 461 | 3.44 |
| 1998 | 160 | 1.16 | 238 | 1.79 | 442 | 3.28 |
| 1999 | 145 | 1.06 | 222 | 1.64 | 407 | 2.98 |
| 2000 | 142 | 1.03 | 212 | 1.54 | 353 | 2.53 |
| 2001 | 161 | 1.16 | 246 | 1.78 | 329 | 2.33 |
| 2002 | 136 | 0.98 | 233 | 1.64 | 310 | 2.19 |
| 2003 | 125 | 0.90 | 236 | 1.65 | 316 | 2.23 |
| 2004 | 142 | 1.01 | 254 | 1.79 | 263 | 1.82 |
| 2005 | 145 | 1.04 | 253 | 1.76 | 248 | 1.71 |
| 2006 | 146 | 1.05 | 283 | 1.94 | 217 | 1.47 |
| 2007 | 140 | 0.98 | 252 | 1.71 | 200 | 1.34 |
| 2008 | 164 | 1.13 | 255 | 1.71 | 206 | 1.35 |
| 2009 | 140 | 0.97 | 284 | 1.88 | 228 | 1.48 |
| 2010 | 133 | 0.92 | 289 | 1.90 | 210 | 1.34 |
| 2011 | 127 | 0.87 | 309 | 2.00 | 203 | 1.34 |
| 2012 | 132 | 0.91 | 301 | 1.89 | 175 | 1.12 |
| 2013 | 120 | 0.82 | 312 | 1.97 | 184 | 1.17 |
| 2014 | 170 | 1.13 | 320 | 2.05 | 186 | 1.15 |
| 2015 | 166 | 1.07 | 344 | 2.12 | 142 | 0.85 |
| 2016 | 163 | 1.08 | 324 | 1.98 | 151 | 0.91 |
| 2017 | 141 | 0.93 | 321 | 1.96 | 135 | 0.82 |
| 2018 | 160 | 1.05 | 330 | 2.03 | 170 | 1.01 |

^a^ Rates were calculated as number of cases per 100,000 person-years and age-adjusted to the 2000 U.S. standard population.

eTable 10. Observed total US cervical cancer mortality and SEER-9 cervical cancer incidence-based mortality rates^a^ during 1995-2018, overall and according to race

| Year of death | Total U.S. | | SEER-9 overall | | Race | | | | | |
| --- | --- | --- | --- | --- | --- | --- | --- | --- | --- | --- |
|  |  |  |  |  | White | | Black | | Other | |
|  | No. deaths | Rate | No. deaths | Rate | No. deaths | Rate | No. deaths | Rate | No. deaths | Rate |
| 1995 | 4503 | 3.24 | 630 | 4.79 | 452 | 4.16 | 124 | 11.18 | 54 | 5.21 |
| 1996 | 4540 | 3.21 | 681 | 5.05 | 513 | 4.60 | 119 | 10.78 | 49 | 4.54 |
| 1997 | 4499 | 3.14 | 695 | 5.10 | 522 | 4.63 | 120 | 10.16 | 53 | 4.72 |
| 1998 | 4340 | 2.98 | 686 | 4.95 | 491 | 4.31 | 121 | 10.18 | 74 | 6.29 |
| 1999 | 4204 | 2.83 | 670 | 4.79 | 506 | 4.41 | 111 | 9.18 | 53 | 4.15 |
| 2000 | 4200 | 2.79 | 710 | 4.93 | 500 | 4.25 | 136 | 10.83 | 74 | 5.65 |
| 2001 | 4092 | 2.67 | 678 | 4.68 | 495 | 4.21 | 124 | 9.81 | 59 | 4.44 |
| 2002 | 3952 | 2.55 | 665 | 4.51 | 493 | 4.12 | 122 | 9.49 | 50 | 3.36 |
| 2003 | 3919 | 2.49 | 726 | 4.85 | 538 | 4.46 | 125 | 9.44 | 63 | 4.16 |
| 2004 | 3850 | 2.42 | 696 | 4.57 | 518 | 4.23 | 113 | 8.22 | 65 | 4.19 |
| 2005 | 3924 | 2.42 | 687 | 4.46 | 512 | 4.19 | 117 | 8.25 | 58 | 3.61 |
| 2006 | 3976 | 2.42 | 707 | 4.56 | 487 | 3.95 | 127 | 9.12 | 93 | 5.52 |
| 2007 | 4021 | 2.42 | 727 | 4.53 | 503 | 3.97 | 145 | 9.73 | 79 | 4.47 |
| 2008 | 4008 | 2.37 | 723 | 4.48 | 525 | 4.14 | 125 | 8.25 | 73 | 3.97 |
| 2009 | 3909 | 2.29 | 685 | 4.17 | 487 | 3.75 | 126 | 7.93 | 72 | 3.80 |
| 2010 | 3939 | 2.26 | 706 | 4.19 | 530 | 4.04 | 113 | 6.85 | 63 | 3.21 |
| 2011 | 4092 | 2.33 | 731 | 4.29 | 516 | 3.90 | 138 | 8.13 | 77 | 3.66 |
| 2012 | 4074 | 2.29 | 772 | 4.53 | 563 | 4.27 | 128 | 7.62 | 81 | 3.79 |
| 2013 | 4217 | 2.33 | 797 | 4.48 | 563 | 4.11 | 141 | 7.81 | 93 | 4.13 |
| 2014 | 4115 | 2.26 | 710 | 3.91 | 498 | 3.58 | 116 | 6.21 | 96 | 4.07 |
| 2015 | 4175 | 2.27 | 732 | 3.99 | 526 | 3.75 | 114 | 6.22 | 92 | 3.80 |
| 2016 | 4188 | 2.24 | 736 | 3.92 | 505 | 3.58 | 137 | 6.73 | 94 | 3.80 |
| 2017 | 4207 | 2.23 | 752 | 3.98 | 542 | 3.77 | 120 | 5.98 | 90 | 3.46 |
| 2018 | 4138 | 2.17 | 797 | 4.20 | 574 | 4.08 | 127 | 6.39 | 96 | 3.49 |

^a^ Based on cases diagnosed during 1975–2018. Rates were calculated as number of deaths per 100,000 person-years and age-adjusted to the 2000 U.S. standard population.

eTable 11. Cervical cancer incidence-based mortality rates^a^ during 1995-2018 according to age group

| Year of death | Age groups | | | | | | | | | |
| --- | --- | --- | --- | --- | --- | --- | --- | --- | --- | --- |
|  | 0-19 | | 20-39 | | 40-59 | | 60-79 | | 80+ | |
|  | No. deaths | Rate | No. deaths | Rate | No. deaths | Rate | No. deaths | Rate | No. deaths | Rate |
| 1995 | - | - | 56 | 1.37 | 179 | 5.90 | 256 | 14.51 | 138 | 27.50 |
| 1996 | - | - | 58 | 1.41 | 174 | 5.50 | 293 | 16.59 | 156 | 30.34 |
| 1997 | - | - | 49 | 1.22 | 201 | 6.12 | 281 | 15.93 | 164 | 31.06 |
| 1998 | - | - | 52 | 1.30 | 206 | 6.02 | 258 | 14.65 | 170 | 31.55 |
| 1999 | - | - | 35 | 0.89 | 212 | 6.03 | 266 | 15.09 | 157 | 28.50 |
| 2000 | - | - | 50 | 1.28 | 206 | 5.70 | 256 | 14.39 | 197 | 34.63 |
| 2001 | - | - | 43 | 1.12 | 192 | 5.17 | 261 | 14.67 | 182 | 31.64 |
| 2002 | - | - | 31 | 0.79 | 201 | 5.22 | 260 | 14.59 | 172 | 29.28 |
| 2003 | - | - | 37 | 1.00 | 224 | 5.71 | 267 | 14.80 | 198 | 33.13 |
| 2004 | - | - | 40 | 1.06 | 206 | 5.13 | 252 | 13.88 | 198 | 32.32 |
| 2005 | - | - | 32 | 0.87 | 228 | 5.64 | 246 | 13.37 | 180 | 28.56 |
| 2006 | - | - | 46 | 1.24 | 221 | 5.22 | 269 | 14.67 | 171 | 26.76 |
| 2007 | - | - | 39 | 1.03 | 204 | 4.74 | 282 | 14.71 | 202 | 31.10 |
| 2008 | - | - | 38 | 1.02 | 210 | 4.81 | 297 | 15.21 | 178 | 27.23 |
| 2009 | - | - | 34 | 0.90 | 200 | 4.69 | 267 | 13.35 | 184 | 27.42 |
| 2010 | - | - | 26 | 0.67 | 204 | 4.73 | 296 | 14.23 | 180 | 25.92 |
| 2011 | - | - | 34 | 0.93 | 231 | 5.28 | 274 | 12.82 | 191 | 27.82 |
| 2012 | - | - | 56 | 1.52 | 215 | 4.78 | 309 | 14.38 | 192 | 28.13 |
| 2013 | - | - | 37 | 1.01 | 239 | 5.45 | 316 | 13.43 | 205 | 29.24 |
| 2014 | - | - | 34 | 0.88 | 202 | 4.56 | 274 | 11.44 | 200 | 28.26 |
| 2015 | - | - | 31 | 0.82 | 174 | 3.97 | 340 | 13.81 | 187 | 26.42 |
| 2016 | - | - | 31 | 0.78 | 193 | 4.37 | 318 | 12.28 | 194 | 27.56 |
| 2017 | - | - | 41 | 1.02 | 179 | 4.23 | 334 | 12.44 | 198 | 27.72 |
| 2018 | - | - | 48 | 1.20 | 210 | 4.78 | 330 | 12.34 | 209 | 29.14 |

^a^ Based on cases diagnosed during 1975–2018. Rates were calculated as number of deaths per 100,000 person-years and age-adjusted to the 2000 U.S. standard population.

-Statistic suppressed due to <16 deaths in the time interval.

eTable 12. Cervical cancer incidence-based mortality rates^a^ during 1995-2018 according to median household income and rural-urban distribution

| Year of death | Median household income | | | | Rural-urban distribution | | | |
| --- | --- | --- | --- | --- | --- | --- | --- | --- |
|  | < $75000 | | ≥ $75000 | | Metropolitan | | Non-metropolitan | |
|  | No. deaths | Rate | No. deaths | Rate | No. deaths | Rate | No. deaths | Rate |
| 1995 | 239 | 1.84 | 150 | 1.16 | 322 | 2.49 | 48 | 0.37 |
| 1996 | 242 | 1.82 | 192 | 1.43 | 361 | 2.69 | 61 | 0.46 |
| 1997 | 258 | 1.93 | 177 | 1.31 | 346 | 2.58 | 68 | 0.50 |
| 1998 | 259 | 1.89 | 218 | 1.58 | 383 | 2.78 | 65 | 0.47 |
| 1999 | 240 | 1.73 | 199 | 1.45 | 359 | 2.60 | 64 | 0.46 |
| 2000 | 244 | 1.72 | 225 | 1.59 | 382 | 2.69 | 60 | 0.43 |
| 2001 | 245 | 1.70 | 226 | 1.58 | 398 | 2.78 | 62 | 0.43 |
| 2002 | 239 | 1.65 | 218 | 1.49 | 383 | 2.63 | 66 | 0.45 |
| 2003 | 308 | 2.09 | 207 | 1.39 | 421 | 2.85 | 84 | 0.56 |
| 2004 | 274 | 1.81 | 239 | 1.58 | 419 | 2.78 | 84 | 0.55 |
| 2005 | 279 | 1.83 | 226 | 1.50 | 433 | 2.86 | 67 | 0.43 |
| 2006 | 295 | 1.92 | 227 | 1.50 | 432 | 2.84 | 80 | 0.51 |
| 2007 | 324 | 2.06 | 217 | 1.37 | 456 | 2.89 | 79 | 0.50 |
| 2008 | 320 | 2.02 | 216 | 1.34 | 455 | 2.85 | 72 | 0.46 |
| 2009 | 338 | 2.10 | 189 | 1.15 | 452 | 2.79 | 71 | 0.44 |
| 2010 | 315 | 1.91 | 199 | 1.18 | 432 | 2.60 | 76 | 0.46 |
| 2011 | 346 | 2.09 | 220 | 1.27 | 500 | 2.96 | 59 | 0.36 |
| 2012 | 378 | 2.29 | 214 | 1.23 | 510 | 3.05 | 77 | 0.44 |
| 2013 | 398 | 2.31 | 217 | 1.22 | 530 | 3.03 | 80 | 0.47 |
| 2014 | 342 | 1.91 | 201 | 1.13 | 472 | 2.64 | 65 | 0.37 |
| 2015 | 370 | 2.06 | 193 | 1.05 | 491 | 2.71 | 63 | 0.34 |
| 2016 | 370 | 2.00 | 214 | 1.15 | 518 | 2.79 | 62 | 0.34 |
| 2017 | 363 | 1.96 | 214 | 1.18 | 499 | 2.72 | 74 | 0.40 |
| 2018 | 381 | 2.08 | 241 | 1.26 | 521 | 2.81 | 93 | 0.50 |

^a^ Based on cases diagnosed during 1990–2018. Rates were calculated as number of deaths per 100,000 person-years and age-adjusted to the 2000 U.S. standard population.

eTable 13. Cervical cancer incidence-based mortality rates^a^ during 1995-2018 according to histologic type

| Year of death | Histologic type | | | | | | | |
| --- | --- | --- | --- | --- | --- | --- | --- | --- |
|  | Squamous cell carcinoma | | Adenocarcinoma | | Adenosquamous carcinoma | | Other | |
|  | No. deaths | Rate | No. deaths | Rate | No. deaths | Rate | No. deaths | Rate |
| 1995 | 452 | 3.45 | 66 | 0.50 | 23 | 0.18 | 89 | 0.67 |
| 1996 | 510 | 3.80 | 63 | 0.46 | 23 | 0.17 | 85 | 0.62 |
| 1997 | 497 | 3.66 | 87 | 0.64 | 21 | 0.15 | 90 | 0.65 |
| 1998 | 480 | 3.48 | 77 | 0.55 | 33 | 0.25 | 96 | 0.67 |
| 1999 | 483 | 3.45 | 76 | 0.55 | 25 | 0.18 | 86 | 0.62 |
| 2000 | 496 | 3.45 | 82 | 0.57 | 29 | 0.21 | 103 | 0.70 |
| 2001 | 461 | 3.18 | 79 | 0.54 | 30 | 0.21 | 108 | 0.74 |
| 2002 | 485 | 3.32 | 70 | 0.47 | 30 | 0.21 | 80 | 0.52 |
| 2003 | 520 | 3.46 | 83 | 0.57 | 20 | 0.13 | 103 | 0.69 |
| 2004 | 476 | 3.12 | 93 | 0.61 | 27 | 0.19 | 100 | 0.65 |
| 2005 | 502 | 3.27 | 79 | 0.51 | 21 | 0.14 | 85 | 0.54 |
| 2006 | 490 | 3.17 | 81 | 0.52 | 27 | 0.17 | 109 | 0.70 |
| 2007 | 494 | 3.06 | 98 | 0.62 | 27 | 0.16 | 108 | 0.68 |
| 2008 | 494 | 3.08 | 98 | 0.59 | 22 | 0.14 | 109 | 0.67 |
| 2009 | 469 | 2.87 | 85 | 0.51 | 20 | 0.12 | 111 | 0.67 |
| 2010 | 498 | 2.93 | 88 | 0.54 | 18 | 0.11 | 102 | 0.61 |
| 2011 | 496 | 2.94 | 86 | 0.50 | 28 | 0.18 | 121 | 0.68 |
| 2012 | 518 | 3.04 | 117 | 0.69 | 29 | 0.17 | 108 | 0.63 |
| 2013 | 542 | 3.04 | 111 | 0.64 | 20 | 0.10 | 124 | 0.70 |
| 2014 | 482 | 2.67 | 103 | 0.55 | 24 | 0.13 | 101 | 0.55 |
| 2015 | 498 | 2.70 | 93 | 0.51 | 23 | 0.13 | 118 | 0.65 |
| 2016 | 495 | 2.64 | 104 | 0.55 | 16 | 0.10 | 121 | 0.63 |
| 2017 | 496 | 2.62 | 116 | 0.63 | 17 | 0.08 | 123 | 0.65 |
| 2018 | 525 | 2.77 | 111 | 0.60 | 21 | 0.11 | 140 | 0.72 |

^a^ Based on cases diagnosed during 1975–2018. Rates were calculated as number of deaths per 100,000 person-years and age-adjusted to the 2000 U.S. standard population.

eTable 14. Squamous cell carcinoma incidence-based mortality rates^a^ during 1995-2017 according to WHO grade

| Year of death | WHO grade | | | | | | | |
| --- | --- | --- | --- | --- | --- | --- | --- | --- |
|  | I | | II | | III | | Unknown | |
|  | No. deaths | Rate | No. deaths | Rate | No. deaths | Rate | No. deaths | Rate |
| 1995 | 32 | 0.25 | 101 | 0.77 | 133 | 1.03 | 186 | 1.40 |
| 1996 | 21 | 0.17 | 126 | 0.95 | 149 | 1.11 | 214 | 1.58 |
| 1997 | 23 | 0.17 | 131 | 0.96 | 145 | 1.09 | 198 | 1.44 |
| 1998 | 19 | 0.14 | 118 | 0.87 | 152 | 1.08 | 191 | 1.39 |
| 1999 | 18 | 0.13 | 116 | 0.83 | 150 | 1.07 | 199 | 1.41 |
| 2000 | 21 | 0.15 | 116 | 0.81 | 140 | 0.99 | 219 | 1.50 |
| 2001 | 19 | 0.13 | 134 | 0.94 | 141 | 0.98 | 167 | 1.14 |
| 2002 | 27 | 0.18 | 127 | 0.87 | 137 | 0.95 | 194 | 1.32 |
| 2003 | 21 | 0.14 | 128 | 0.85 | 166 | 1.13 | 205 | 1.34 |
| 2004 | 23 | 0.14 | 131 | 0.87 | 143 | 0.95 | 179 | 1.16 |
| 2005 | 19 | 0.12 | 131 | 0.85 | 149 | 0.97 | 203 | 1.34 |
| 2006 | 28 | 0.19 | 123 | 0.82 | 146 | 0.94 | 193 | 1.23 |
| 2007 | 19 | 0.12 | 134 | 0.85 | 137 | 0.84 | 204 | 1.26 |
| 2008 | 24 | 0.14 | 143 | 0.89 | 151 | 0.96 | 176 | 1.09 |
| 2009 | 21 | 0.12 | 117 | 0.71 | 160 | 1.02 | 171 | 1.02 |
| 2010 | 25 | 0.15 | 111 | 0.66 | 154 | 0.91 | 208 | 1.22 |
| 2011 | 20 | 0.11 | 148 | 0.88 | 150 | 0.91 | 178 | 1.04 |
| 2012 | 26 | 0.14 | 140 | 0.83 | 148 | 0.90 | 204 | 1.17 |
| 2013 | 17 | 0.09 | 158 | 0.90 | 168 | 0.96 | 199 | 1.09 |
| 2014 | - | - | 141 | 0.80 | 144 | 0.82 | 184 | 0.98 |
| 2015 | 19 | 0.10 | 124 | 0.70 | 146 | 0.79 | 209 | 1.12 |
| 2016 | 16 | 0.08 | 123 | 0.69 | 140 | 0.76 | 216 | 1.11 |
| 2017 | 20 | 0.11 | 112 | 0.60 | 143 | 0.78 | 221 | 1.13 |

^a^ Based on cases diagnosed during 1975–2017. Rates were calculated as number of deaths per 100,000 person-years and age-adjusted to the 2000 U.S. standard population.

-Statistic suppressed due to <16 deaths in the time interval.

eTable 15. Squamous cell carcinoma incidence-based mortality rates^a^ during 1995-2018 according to stage

| Year of death | Stage | | | | | | | |
| --- | --- | --- | --- | --- | --- | --- | --- | --- |
|  | Localized | | Regional | | Distant | | Unknown | |
|  | No. deaths | Rate | No. deaths | Rate | No. deaths | Rate | No. deaths | Rate |
| 1995 | 138 | 1.04 | 220 | 1.68 | 48 | 0.39 | 46 | 0.35 |
| 1996 | 175 | 1.30 | 253 | 1.91 | 40 | 0.30 | 42 | 0.30 |
| 1997 | 188 | 1.37 | 229 | 1.69 | 55 | 0.42 | 25 | 0.18 |
| 1998 | 172 | 1.23 | 232 | 1.70 | 47 | 0.35 | 29 | 0.21 |
| 1999 | 188 | 1.34 | 219 | 1.56 | 44 | 0.33 | 32 | 0.22 |
| 2000 | 205 | 1.40 | 213 | 1.49 | 44 | 0.32 | 34 | 0.24 |
| 2001 | 183 | 1.25 | 202 | 1.40 | 57 | 0.40 | 19 | 0.13 |
| 2002 | 199 | 1.36 | 202 | 1.39 | 64 | 0.44 | 20 | 0.14 |
| 2003 | 216 | 1.43 | 218 | 1.44 | 59 | 0.42 | 27 | 0.17 |
| 2004 | 186 | 1.20 | 211 | 1.39 | 56 | 0.38 | 24 | 0.16 |
| 2005 | 183 | 1.19 | 222 | 1.44 | 79 | 0.54 | 27 | 0.17 |
| 2006 | 180 | 1.14 | 218 | 1.43 | 81 | 0.53 | 21 | 0.14 |
| 2007 | 202 | 1.26 | 204 | 1.25 | 65 | 0.42 | 28 | 0.16 |
| 2008 | 197 | 1.19 | 232 | 1.47 | 58 | 0.37 | 16 | 0.11 |
| 2009 | 189 | 1.13 | 193 | 1.21 | 71 | 0.44 | 23 | 0.13 |
| 2010 | 211 | 1.24 | 216 | 1.28 | 63 | 0.38 | 21 | 0.11 |
| 2011 | 182 | 1.05 | 216 | 1.31 | 88 | 0.54 | 21 | 0.11 |
| 2012 | 203 | 1.16 | 219 | 1.31 | 87 | 0.54 | 23 | 0.12 |
| 2013 | 225 | 1.23 | 230 | 1.29 | 85 | 0.51 | - | - |
| 2014 | 201 | 1.09 | 202 | 1.13 | 72 | 0.42 | - | - |
| 2015 | 221 | 1.14 | 204 | 1.14 | 68 | 0.39 | 16 | 0.09 |
| 2016 | 199 | 1.04 | 204 | 1.10 | 90 | 0.50 | 16 | 0.08 |
| 2017 | 207 | 1.06 | 189 | 1.01 | 82 | 0.46 | 21 | 0.11 |
| 2018 | 231 | 1.18 | 200 | 1.10 | 83 | 0.44 | - | - |

^a^ Based on cases diagnosed during 1975–2018. Rates were calculated as number of deaths per 100,000 person-years and age-adjusted to the 2000 U.S. standard population.

-Statistic suppressed due to <16 deaths in the time interval.

eTable 16. Squamous cell carcinoma incidence-based mortality rates^a^ during 1995-2018 according to AJCC stage

| Year of death | AJCC stage | | | | | | | | | |
| --- | --- | --- | --- | --- | --- | --- | --- | --- | --- | --- |
|  | I | | II | | III | | IV | | Unknown | |
|  | No. deaths | Rate | No. deaths | Rate | No. deaths | Rate | No. deaths | Rate | No. deaths | Rate |
| 1995 | 57 | 0.43 | 63 | 0.49 | 80 | 0.62 | 54 | 0.43 | 30 | 0.23 |
| 1996 | 87 | 0.64 | 76 | 0.57 | 99 | 0.77 | 46 | 0.35 | 36 | 0.26 |
| 1997 | 84 | 0.63 | 72 | 0.54 | 78 | 0.59 | 60 | 0.46 | 24 | 0.17 |
| 1998 | 102 | 0.74 | 68 | 0.50 | 90 | 0.66 | 59 | 0.44 | 23 | 0.17 |
| 1999 | 97 | 0.70 | 68 | 0.49 | 69 | 0.51 | 53 | 0.40 | 27 | 0.19 |
| 2000 | 106 | 0.74 | 60 | 0.43 | 76 | 0.54 | 52 | 0.37 | 26 | 0.18 |
| 2001 | 100 | 0.70 | 69 | 0.47 | 69 | 0.49 | 67 | 0.47 | - | - |
| 2002 | 124 | 0.86 | 53 | 0.37 | 83 | 0.59 | 62 | 0.43 | - | - |
| 2003 | 143 | 0.96 | 72 | 0.48 | 78 | 0.53 | 64 | 0.45 | 18 | 0.12 |
| 2004 | 117 | 0.77 | 67 | 0.44 | 82 | 0.55 | 63 | 0.43 | 23 | 0.15 |
| 2005 | 119 | 0.79 | 71 | 0.47 | 84 | 0.55 | 78 | 0.53 | 31 | 0.19 |
| 2006 | 117 | 0.75 | 80 | 0.53 | 79 | 0.53 | 78 | 0.51 | 24 | 0.15 |
| 2007 | 143 | 0.90 | 72 | 0.44 | 69 | 0.44 | 65 | 0.42 | 26 | 0.15 |
| 2008 | 120 | 0.74 | 74 | 0.48 | 99 | 0.62 | 57 | 0.37 | 22 | 0.15 |
| 2009 | 129 | 0.79 | 74 | 0.47 | 76 | 0.48 | 67 | 0.42 | 26 | 0.16 |
| 2010 | 131 | 0.78 | 64 | 0.37 | 92 | 0.56 | 59 | 0.36 | 24 | 0.13 |
| 2011 | 117 | 0.69 | 65 | 0.41 | 99 | 0.61 | 86 | 0.52 | 24 | 0.13 |
| 2012 | 128 | 0.74 | 80 | 0.47 | 89 | 0.55 | 84 | 0.52 | 26 | 0.14 |
| 2013 | 145 | 0.82 | 72 | 0.41 | 98 | 0.56 | 81 | 0.49 | 24 | 0.13 |
| 2014 | 127 | 0.71 | 69 | 0.37 | 90 | 0.53 | 71 | 0.41 | 18 | 0.10 |
| 2015 | 152 | 0.80 | 70 | 0.39 | 85 | 0.49 | 66 | 0.38 | 22 | 0.13 |
| 2016 | 137 | 0.72 | 65 | 0.34 | 87 | 0.50 | 88 | 0.49 | 16 | 0.08 |
| 2017 | 127 | 0.66 | 60 | 0.32 | 99 | 0.56 | 80 | 0.45 | 21 | 0.11 |
| 2018 | 153 | 0.78 | 46 | 0.24 | 116 | 0.67 | 86 | 0.46 | 17 | 0.09 |

Abbreviation: AJCC, American Joint Committee on Cancer

^a^ Based on cases diagnosed during 1988–2018. Rates were calculated as number of deaths per 100,000 person-years and age-adjusted to the 2000 U.S. standard population.

-Statistic suppressed due to <16 deaths in the time interval.

eTable 17. Squamous cell carcinoma incidence-based mortality rates^a^ during 1995-2018 according to tumor size

| Year of death | Tumor size | | | | | |
| --- | --- | --- | --- | --- | --- | --- |
|  | < 2cm | | ≥ 2cm | | Unknown | |
|  | No. deaths | Rate | No. deaths | Rate | No. deaths | Rate |
| 1995 | - | - | 113 | 0.90 | 224 | 1.72 |
| 1996 | - | - | 120 | 0.89 | 270 | 2.03 |
| 1997 | 17 | 0.13 | 110 | 0.83 | 251 | 1.86 |
| 1998 | 20 | 0.15 | 132 | 0.98 | 234 | 1.70 |
| 1999 | 29 | 0.21 | 110 | 0.80 | 231 | 1.66 |
| 2000 | 23 | 0.16 | 113 | 0.81 | 254 | 1.77 |
| 2001 | 18 | 0.12 | 108 | 0.77 | 236 | 1.62 |
| 2002 | 28 | 0.20 | 122 | 0.86 | 241 | 1.65 |
| 2003 | 27 | 0.18 | 137 | 0.93 | 258 | 1.72 |
| 2004 | 26 | 0.18 | 138 | 0.94 | 242 | 1.56 |
| 2005 | 40 | 0.27 | 151 | 1.01 | 228 | 1.48 |
| 2006 | 31 | 0.21 | 146 | 0.98 | 235 | 1.51 |
| 2007 | 33 | 0.21 | 147 | 0.94 | 239 | 1.46 |
| 2008 | 35 | 0.21 | 164 | 1.04 | 216 | 1.35 |
| 2009 | 27 | 0.17 | 146 | 0.93 | 234 | 1.41 |
| 2010 | 30 | 0.17 | 180 | 1.11 | 211 | 1.21 |
| 2011 | 44 | 0.25 | 162 | 1.02 | 220 | 1.29 |
| 2012 | 42 | 0.24 | 191 | 1.16 | 215 | 1.23 |
| 2013 | 41 | 0.24 | 199 | 1.18 | 222 | 1.21 |
| 2014 | 38 | 0.21 | 181 | 1.03 | 195 | 1.08 |
| 2015 | 44 | 0.23 | 181 | 1.02 | 207 | 1.10 |
| 2016 | 28 | 0.15 | 203 | 1.15 | 206 | 1.04 |
| 2017 | 39 | 0.22 | 200 | 1.10 | 190 | 0.99 |
| 2018 | 55 | 0.28 | 208 | 1.17 | 186 | 0.93 |

^a^ Based on cases diagnosed during 1983–2018. Rates were calculated as number of deaths per 100,000 person-years and age-adjusted to the 2000 U.S. standard population.

-Statistic suppressed due to <16 deaths in the time interval.
